# Bulk and single-cell transcriptomics reveal prognostic signatures of phosphoinositide metabolism in lung adenocarcinoma

**DOI:** 10.1038/s41598-026-56684-2

**Published:** 2026-06-10

**Authors:** Lihua Zhou, Peng Lei, Zhouguang Luo, Jie Xiao, Zongyu Chen

**Affiliations:** 1 Department of Pulmonary and Critical Care Medicine, Affiliated Hospital of Guizhou Medical University, No. 28 Guiyi Street, Yunyan District, Guiyang, 550000 China; 2 Department of Neurosurgery, Affiliated Hospital of Guizhou Medical University, No. 28 Guiyi Street, Yunyan District, Guiyang, 550000 China; 3 Department of Infectious Disease, Longgang People’s Hospital (The Longgang Branch of the First Affiliated Hospital of Wenzhou Medical University), No.980-1208 New City Avenue, Longgang, 325802 China; 4 Department of Emergency, Affiliated Hospital of Guizhou Medical University, No. 28 Guiyi Street, Yunyan District, Guiyang, 550000 China

**Keywords:** Lung adenocarcinoma, Phosphoinositides metabolism, Single-cell transcriptomics, Prognostic genes, Risk model, Biomarkers, Cancer, Computational biology and bioinformatics, Oncology

## Abstract

Lung adenocarcinoma (LUAD) is one of the most severe malignant tumors. Phosphoinositides metabolism (PIM) plays an important role in maintaining the normal life activities of the organism and regulating tumor development. This study aimed to comprehensively investigate the association between LUAD and PIM. Data on LUAD and PIM-related genes (PIM-RGs) were sourced from public databases. Differential expression, univariate Cox regression analyses, and machine learning were conducted to identify prognostic genes. A risk model was subsequently developed, and LUAD patients were categorized into a high-risk group (HRG) and a low-risk group (LRG). Independent prognostic factors for LUAD were identified, and a nomogram was constructed. Functional enrichment, tumor microenvironment, mutations, and drug sensitivity analyses were also conducted to investigate the molecular mechanisms underlying LUAD. Additionally, single-cell RNA sequencing (scRNA-seq) data analysis was employed to identify key cells and clarify the dynamics of prognostic genes’ expression. Ultimately, prognostic gene expression was investigated in clinical samples. MTMR7, GDPD1, MTMR4, and MTMR10 were recognized as prognostic genes. The risk model and nomogram (incorporating risk score and Stage) had good predictive performance. Notably, LUAD’s malignant progression might be closely associated with biological processes including cellular protein synthesis, abnormal activation of neuroactive ligand-receptor interactions, and anti-tumor immune responses. Additionally, there was a general positive correlation between the differentially infiltrated immune cells of HRG and LRG. Moreover, TP53 and TTN had relatively high mutation frequencies in both HRG and LRG, and 142 drugs exhibited differential sensitivity between the 2 groups. Interestingly, epithelial cells were identified as LUAD’s key cell type, with prognostic gene expression showing dynamic changes as these cells differentiated. Consistently, compared with the control group, GDPD1 and MTMR4 were upregulated, MTMR10 was downregulated, and MTMR7 showed no statistical difference but a certain upward trend in the LUAD group. This study identified 4 prognostic genes and constructed an effective risk model, providing a new perspective on the treatment of LUAD.

**Supplementary Information:**

The online version contains supplementary material available at 10.1038/s41598-026-56684-2.

## Introduction

Lung adenocarcinoma (LUAD), the most prevalent subtype of non-small cell lung cancer (NSCLC), ranks among the leading causes of cancer-related mortality globally^1^. The primary therapeutic modalities for advanced lung cancer currently encompass radiotherapy, chemotherapy, targeted therapy, and immunotherapy. Despite advancements in these treatments, considerable variability in patient outcomes remains, primarily due to tumor heterogeneity and mechanisms of drug resistance^2–4^. The prognostic accuracy of the existing TNM staging system is limited^5^, underscoring the urgent need for novel biomarkers to enhance risk stratification and facilitate personalized treatment approaches.

Phosphoinositides metabolism (PIM) represents a conserved and fundamental intracellular metabolic pathway that orchestrates critical cellular processes, including membrane trafficking, signal transduction, and cell proliferation, through the dynamic regulation of phosphoinositides^6,7^. Notably, the PI3K/AKT/mTOR signaling cascade, a downstream pathway of PIM, is often dysregulated in lung adenocarcinoma (LUAD), facilitating malignant progression and contributing to resistance against chemotherapy and targeted therapies^8,9^. The advent of single-cell sequencing technology permits a high-resolution examination of cellular heterogeneity within the LUAD tumor microenvironment, thereby providing valuable insights into disease mechanisms and identifying potential therapeutic targets^10–12^. Consequently, a systematic investigation into the molecular interactions between PIM and LUAD, coupled with the identification of novel PIM-related prognostic genes and an in-depth exploration of LUAD heterogeneity at the cellular level, will not only advance the understanding of the pathological molecular mechanisms underlying LUAD but also lay a theoretical foundation for the development of precise therapeutic targets and the optimization of clinical treatment strategies.

This study employed lung adenocarcinoma (LUAD) data and PIM-related genes (PIM-RGs) sourced from online databases, integrating bioinformatics approaches to identify genes significantly associated with LUAD prognosis and to develop a risk model. To systematically assess the applicability of these prognostic genes and the constructed risk model, independent prognostic analyses were performed to validate their predictive accuracy. Furthermore, functional enrichment analysis, tumor microenvironment analysis, somatic mutation analysis, and drug sensitivity analysis were conducted to elucidate their potential roles in the pathogenesis of LUAD. Additionally, single-cell analysis was utilized to uncover key features of cellular heterogeneity and expression patterns of prognostic molecules at the cellular level in LUAD, offering a refined perspective for mechanistic investigations.

## Materials and methods

### Data acquisition

A total of 4 datasets related to lung adenocarcinoma (LUAD) were used in this study. To elaborate, the TCGA-LUAD was downloaded from The Cancer Genome Atlas (TCGA) database (https://portal.gdc.cancer.gov/) on July 8, 2025, and assigned as the training set for this research. The TCGA-LUAD comprised 513 tumor tissue samples (LUAD group), of which 440 had complete survival information (overall survival, OS), and 58 control lung tissue samples (control group). Additionally, 3 datasets, GSE50081 (platform: GPL570), GSE72094 (platform: GPL15048), and the single-cell RNA sequencing (scRNA-seq) dataset GSE131907 (platform: GPL16791), were downloaded from the Gene Expression Omnibus (GEO) database (https://www.ncbi.nlm.nih.gov/geo/). In this research, only LUAD tissue samples with complete survival information were selected for the evaluation of the risk model, specifically including 127 samples from GSE50081 and 398 samples from GSE72094 (both datasets downloaded on July 9, 2025). Other disease samples in these 2 datasets were not included in this study. For GSE131907 (downloaded on July 13, 2025), 11 early-stage LUAD tumor tissue samples and 4 late-stage LUAD tumor tissue samples were selected to form the LUAD group, while 11 distant normal lung tissue samples were chosen as the control group. The remaining samples in this dataset were not included in the analysis. Furthermore, the gene set file “REACTOME_PI_METABOLISM.v2023.2.Hs.gmt” was retrieved from the Molecular Signatures Database (MSigDB) (https://www.gsea-msigdb.org/gsea/msigdb), and 84 phosphoinositides metabolism (PIM)-related genes (PIM-RGs) were obtained from it (Table S1). This gene set file was downloaded on July 9, 2025.

### Discernment and related functional analyses of candidate genes

To obtain genes associated with PIM in LUAD, first, the identification of differentially expressed genes (DEGs) comparing the LUAD group against the control group in TCGA-LUAD was accomplished utilizing the DESeq2 package (v 1.40.2)^13^, with the threshold of p.adjust < 0.05 and |log_2_fold change (FC)|> 0.5. Furthermore, a volcano plot for DEGs was generated utilizing the ggplot2 package (v 3.5.2)^14^, and labeled with the top 5 gene names demonstrating significant up- or down-regulation according to the descending order of log_2_FC, while a heatmap of the labeled DEGs was plotted utilizing the ComplexHeatmap package (v 2.16.0)^15^. The intersection of DEGs and PIM-RGs was determined utilizing the ggvenn package (v 0.1.10) (https://www.rdocumentation.org/packages/ggvenn/versions/0.1.10), thereby identifying genes pertinent to PIM within LUAD, which were subsequently designated as candidate genes. Thereafter, Gene Ontology (GO) and Kyoto Encyclopedia of Genes and Genomes (KEGG)^16–18^ analyses (*p* < 0.05) of candidate genes were implemented utilizing the clusterProfiler package (v 4.10.1)^19^, to elucidate biological functions in which the candidate genes were involved. The top 5 terms for each GO category (biological processes (BPs), molecular functions (MFs), and cellular components (CCs), individually) and the top 5 KEGG pathways were displayed, based on the highest number of enriched genes. Moreover, to characterize the interaction network of proteins encoded by candidate genes, Search Tool for the Retrieval of Interacting Genes (STRING) website (https://www.string-db.org) was utilized to characterize a protein–protein interaction (PPI) network (interaction scores > 0.4) (access date: July 14, 2025). After removing the isolated targets, the circlize package (v 0.4.10)^20^ was used to visualize the portion of the results with interactions.

### Identification of prognostic genes and construction of the risk model

In the TCGA-LUAD, candidate genes were subjected to univariate Cox regression analysis employing the survival package (v 3.8–3) (https://www.rdocumentation.org/packages/survival/versions/3.8-3), with the aim of identifying genes exhibiting significant associations with LUAD survival outcomes (hazard ratio (HR) ≠ 1, *p* < 0.05). Results were visualized using the forestplot package (v 2.0.1)^21^. Subsequently, survival-associated genes were subjected to a test for the proportional hazards (PH) assumption (*p* > 0.05), utilizing the cox.zph and ggcoxzph functions. Genes that passed the PH assumption test were included in the tenfold cross-validated least absolute shrinkage and selection operator (LASSO) regression analysis, performed utilizing the glmnet package (v 4.1–8)^22^. At the optimal lambda value, the LASSO model attained the lowest error rate, thereby identifying genes with non-zero regression coefficients as prognostic genes. After that, a risk model was formulated using the prognostic genes, where the risk score was derived based on the ensuing formula:

$$risk score = \mathop \sum \limits_{i = 1}^{n} coef \left( i \right) \times expr\left( i \right)$$

In this equation, coef represented the regression coefficient of each prognostic gene in LASSO analysis, while expr denoted the corresponding expression level of the gene. Thereafter, utilizing the optimal cut-off value for the risk score, 440 LUAD samples from the TCGA-LUAD with comprehensive survival information were stratified into a high-risk group (HRG) and a low-risk group (LRG). Specifically, the risk curve was displayed to illustrate the variation in risk score and survival state distributions between individuals in the HRG and LRG. Subsequently, Kaplan–Meier (K-M) curves were generated for patients in the HRG and LRG using the survminer package (v 0.5.0) (https://www.rdocumentation.org/packages/survminer/versions/0.5.0). The disparity in survival outcomes between these 2 groups was then subjected to a subsequent comparison employing the log-rank test (*p* < 0.05). Furthermore, receiver operating characteristic (ROC) curves for 1-, 3-, and 5-year were plotted employing the survivalROC package (v 1.0.3.1)^23^, with corresponding area under curve (AUC) values being calculated (AUC > 0.6). Eventually, the risk model was validated in GSE50081 and GSE72094 utilizing methods consistent with those utilized in TCGA-LUAD to evaluate its generalisability.

### Independent prognostic analysis and construction of the nomogram

To evaluate and validate the independent predictive ability of the risk score and clinical features for patients, univariate and multivariate Cox regression analyses were performed utilizing the survminer package (v 0.5.0) in the TCGA-LUAD. These analyses aimed to assess whether the risk score, age, gender, Stage, and T/N stages were independent prognostic factors for LUAD. Specifically, univariate Cox regression analysis (*p* < 0.05) was first applied to select prognostic factors. After that, the PH assumption test (*p* > 0.05) was carried out. Multivariate Cox regression analysis was then utilized to pick the final independent prognostic factors. Furthermore, the regplot package (v 1.1)^24^ was used to construct a nomogram model for predicting the 1-, 3-, and 5-year survival probabilities of LUAD patients. In the nomogram, each independent prognostic factor was assigned a unique point; the higher the cumulative sum of these points, the lower the survival rate of LUAD patients. Furthermore, the rms package (v 6.8–1) (https://www.rdocumentation.org/packages/rms/versions/6.8-1) was used to plot calibration curves for evaluating the accuracy of the nomogram. The closer the slopes of the respective calibration curves were to 1, the higher the predictive accuracy of the nomogram was. Meanwhile, decision curve analysis (DCA) was conducted using the rms package (v 6.8–1) to further evaluate the clinical utility of the nomogram at 1-, 3-, and 5-year.

### Gene set enrichment analysis (GSEA)

GSEA was performed to elucidate the differences in biological functions between the HRG and LRG during the progression of LUAD. Initially, DEGs in the HRG and LRG were identified utilizing the DESeq2 package (v 1.40.2), and the obtained genes were then sorted in descending order based on their log_2_FC values. The reference gene set utilized was designated “c2.cp.kegg_legacy.v2025.1.Hs.symbols” from the MSigDB. After that, GSEA was performed utilizing the clusterProfiler package (v 4.10.1) with thresholds of |normalized enrichment score (NES)|> 1, false discovery rate (FDR) < 0.25, and *p* < 0.05. The top 5 most significant pathways were visualized based on *p* values.

### Tumor microenvironment analysis

The ssGSEA algorithm was applied to LUAD samples with complete survival information from TCGA-LUAD using the GSVA package (v 1.46.0)^25^ to calculate the enrichment scores of 28 immune cell types^26^ in the HRG and LRG, and a heatmap was plotted for visualization. Subsequently, the Wilcoxon test was utilized to compare the differences in immune cell infiltration levels between the HRG and LRG (*p* < 0.05). Immune cells with significantly different infiltration abundances were screened and named differentially infiltrated immune cells, which were then visualized using the ggplot2 package (v 3.5.2). To examine the correlations among differentially infiltrated immune cells and between prognostic genes and these cells, Spearman correlation analyses were performed utilizing the psych package (v 2.2.9) (https://www.rdocumentation.org/packages/psych/versions/2.2.9) (|correlation coefficient (cor)|> 0.3, *p* < 0.05). Tumor Immune Dysfunction and Exclusion (TIDE) is a computational tool used to predict immunotherapy responses and evaluate the capacity of tumors for immune evasion. The TIDE database (http://tide.dfci.harvard.edu/) was utilized to obtain TIDE scores of LUAD patients in the HRG and LRG (access date: July 11, 2025). The higher the score, the greater the likelihood of immune evasion. Furthermore, the psych package (v 2.2.9) was used to analyze the Spearman correlation between the TIDE scores and the risk scores, and the Wilcoxon rank-sum test was applied to analyze differences in TIDE scores between the HRG and LRG (*p* < 0.05). Besides, the Wilcoxon test was used to analyze the expression differences of 36 common immune checkpoint genes (ICGs)^27^ (Table S2) included in the TCGA-LUAD between the HRG and LRG, with the significance level set at *p* < 0.05.

### Somatic mutation analysis

Somatic mutations play a crucial role in tumor initiation and progression. To further explore the differences in somatic mutations between the HRG and LRG, the maftools package (v 2.18.0)^28^ was used to analyze somatic mutation data of LUAD samples with survival information in TCGA-LUAD. The tumor mutation burden (TMB) of each sample was calculated and the waterfall plots for the HRG and LRG were generated separately, and genes with the top 20 mutation frequencies were selected for display.

### Drug sensitivity analysis

Chemotherapy is a prevalent therapeutic approach for the management of malignant tumors, employing drugs characterized by a low half-maximal inhibitory concentration (IC50) to effectively suppress cell proliferation. A lower IC50 value denotes heightened sensitivity of the tumor to the drug, thereby enhancing the efficacy of cancer treatment. Sensitivity data for drugs targeting lung adenocarcinoma (LUAD) were obtained from the Genomics of Drug Sensitivity in Cancer (GDSC) database (https://www.cancerrxgene.org/) as of July 11, 2025. IC50 values for these drugs were computed for each LUAD sample derived from the TCGA-LUAD dataset, utilizing the pRRophetic package (v 0.5)^29^. The Wilcoxon test (*p* < 0.05) was applied to assess the differences in IC50 values between high-risk group (HRG) and low-risk group (LRG), and the ggplot2 package (version 3.5.2) was employed to visualize the top five drugs exhibiting significantly different IC50 values between HRG and LRG.

### Identification of the key cell type through scRNA-seq analysis

An extensive investigation was conducted on the scRNA seq data from GSE131907 dataset to explore the underlying mechanisms of LUAD progression. Initially, the key cell type involved in the progression of LUAD was explored. Specifically, the Seurat package (v 5.30)^30^ was applied to process the scRNA-seq data and create Seurat objects. Rigorous quality control (QC) criteria were applied to retain high-quality cells (200 < number of RNA features < 3000, 500 < total RNA count < 10,000, proportion of mitochondrial RNA < 10%). Thereafter, the NormalizeData function was utilized to standardize the data. The top 2000 highly variable genes (HVGs) were identified using the variance-stabilizing transformation (vst) method in the FindVariableFeatures function. Subsequently, the LabelPoints function was used to visualize the results and label the top 10 genes with the greatest expression variability. After that, the data were scaled utilizing the ScaleData function. Furthermore, based on the top 2000 HVGs, principal component analysis (PCA) was performed on the processed GSE131907 using the RunPCA function. To identify highly significant principal components (PCs), the JackStraw function was employed to select PCs enriched for genes with low *p* values (*p* < 0.05). Concomitantly, a scree plot was generated with the ElbowPlot function to ascertain the optimal number of PCs for dimensionality reduction.

Subsequently, unsupervised clustering analysis was performed using the FindNeighbors and FindClusters functions. Cells were clustered via the t-distributed stochastic neighbor embedding (t-SNE) method, and the results were visualized (resolution = 0.4). Following this, the cell clusters were classified into distinct types, referencing marker genes from the literature^31,32^. The RunTSNE function was used to generate t-SNE plots for visualizing. Meanwhile, dot plots were generated to show the expression of marker genes in each cell cluster. Concurrently, the proportions of each cell type in different groups were compared. Afterwards, the expression of prognostic genes in different cell types was analyzed, and the cells with relatively high overall expression levels of prognostic genes were regarded as the key cell type.

#### Pseudo-temporal trajectory and cellular communication analyses

In order to comprehensively analyze the heterogeneity of key cells, the key cell type was subjected to batch effect removal, dimensionality reduction (based on PCs with *p* < 0.05), and clustering (resolution = 0.1) again. The monocle package (v 2.30.1)^33^ was utilized to predict the differentiation states of the key cell type and map their differentiation trajectories. Meanwhile, a heatmap was generated to describe the dynamic trends of the expression of multiple prognostic genes during the differentiation of the key cell type. Additionally, the CellChat package (v 1.6.1)^34^ was utilized to analyze the interaction dynamics among annotated cells in the different groups, revealing the weight and strength of interactions between the key cell type and other annotated cells within each group. After that, the ligand-receptor interaction pairs between cells in different groups from the GSE131907 were also analyzed, and the ggplot2 package (v. 3.5.2) was used to generate bubble plots for visualizing the results.

#### Enrichment and metabolic analyses of the key cell type

To investigate the biological functions involved in the key cell type, the ReactomeGSA package (v 3.20)^35^ was used to explore the functional enrichment and involved biological pathways of the key cell in all samples from GSE131907, and the top 10 most significantly enriched pathways with *p* < 0.05 were selected for presentation. To further understand the metabolic state of the key cell, the VISION algorithm in the scMetabolism package (v 0.2.1)^36^ was applied to conduct metabolic activity analysis of the key cell, and the DotPlot.metabolism function was used to visualize the top 10 significantly differential metabolic pathways (*p* < 0.05).

#### Clinical trial validation of prognostic genes

To investigate the expression of prognostic genes in LUAD, reverse transcription quantitative PCR (RT-qPCR) analysis was performed on clinical samples. Specifically, 12 tissue samples (6 control samples and 6 LUAD samples) were collected from the clinic in the Affiliated Hospital of Guizhou Medical University. Informed consent was obtained from all participants. The study had been reviewed and approved by the Ethics Committee of the Affiliated Hospital of Guizhou Medical University. Total RNA was extracted from the 12 tissue samples using the TRIzol reagent (Vazyme, China) following the manufacturer’s instructions. Subsequently, the RNA concentration was measured with the NanoPhotometer N50. The cDNA was synthesized by reverse transcription using the HP All-in-one qRT Master Mix II RT203-Ver.1 (YoungGen, China), and the reverse transcription was performed with the S1000™ Thermal Cycler (Bio-Rad, USA). The sequences of all primers can be found in Table 1. The qPCR assay was conducted using the CFX Connect Real-time Quantitative Fluorescence PCR Instrument (Bio-Rad, USA). The relative quantification of mRNAs was determined by the 2^-ΔΔCT^ method. The RT-qPCR results were then analyzed and visualized in GraphPad Prism 10 (https://www.graphpad.com/).

**Table 1:** Primer sequences for prognostic genes and GAPDH.

Primer	Sequence (5′-3′)
MTMR7 F	CCGTACGCCCAAGGTTGAAA
MTMR7 R	ACACGTCGTGGCAATCTCTT
GDPD1 F	CTTGCCCTTTGTGCCCATTC
GDPD1 R	TGGTGAGCTATGCTGCTTCC
MTMR4 F	GGAGACGGAAGTGGCAAGAG
MTMR4 R	ACCCATTCTACCGTGGCAAG
MTMR10 F	GGCACCATGTTCTCCCTCAA
MTMR10 R	CTGCTTCCTCTTGTGGTCGT
GAPDH F	ATGGGCAGCCGTTAGGAAAG
GAPDH R	AGGAAAAGCATCACCCGGAG

#### Statistical analysis

Statistical analysis was executed utilizing R (v 4.3.3). The Wilcoxon test was employed to perform differential analyses between the 2 groups, with significance set at *p* < 0.05. Notably, **** represented *p* < 0.0001, *** represented *p* < 0.001, ** represented *p* < 0.01, * represented *p* < 0.05, and no significance (ns) represented *p* > 0.05.

## Results

### Discovery of 36 candidate genes and investigation into their biological roles

**Fig. 1:**
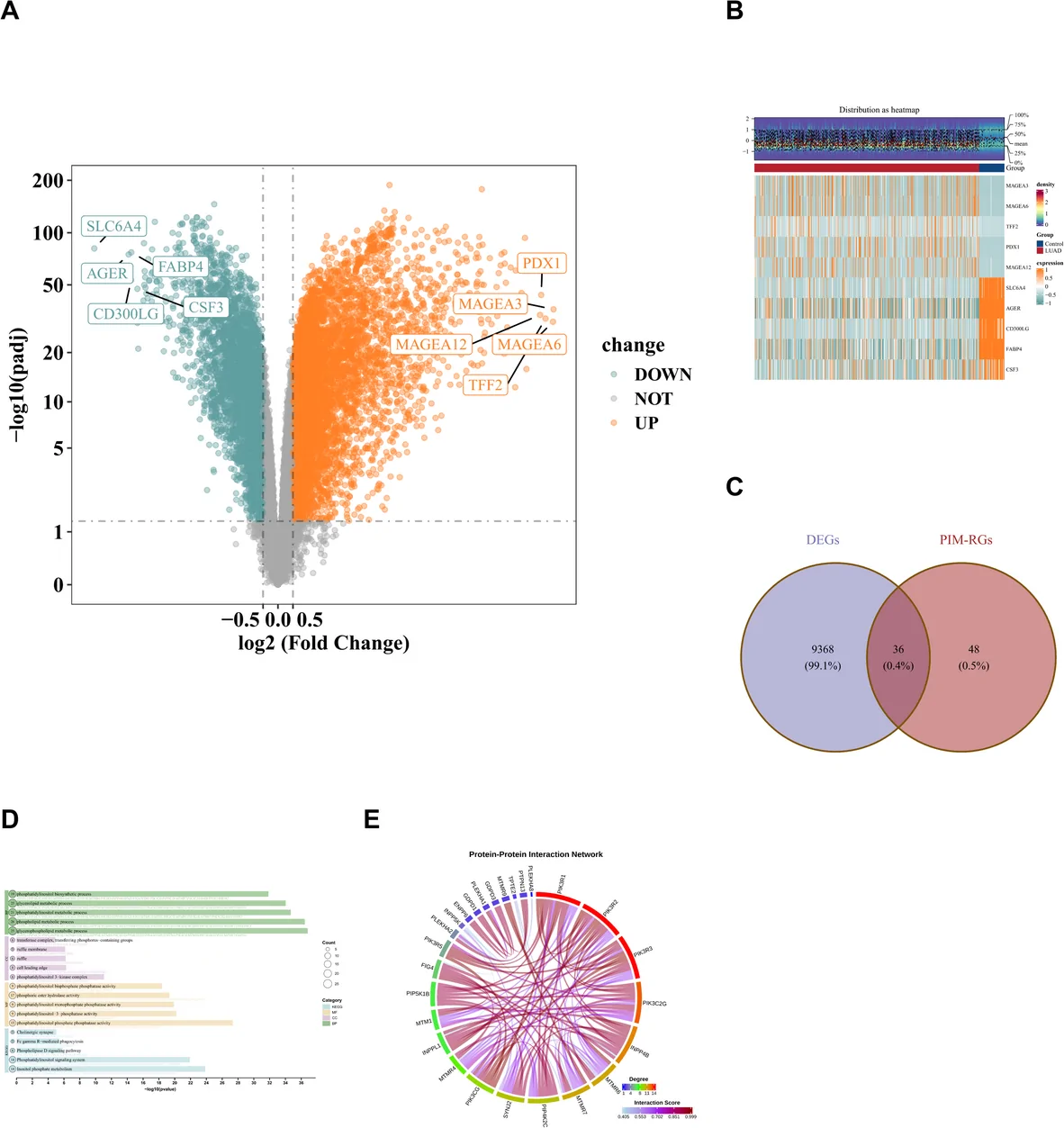
Discovery of 36 candidate genes and investigation into their biological roles. (**A**) and (**B**) The volcano plot and heatmap of DEGs between the LUAD and control groups in TCGA-LUAD; (**C**) Venn of DEGs and PIM-RGs; (**D**) GO and KEGG analyses of candidate genes. The Kyoto Encyclopedia of Genes and Genomes (KEGG) pathway map was obtained from KEGG (https://www.kegg.jp/). KEGG is a publicly available resource under the terms of the academic uselicense^16–18^; (**E**) PPI network of candidate genes.

To identify genes associated with phosphoinositide metabolism (PIM) that exhibited altered expression in lung adenocarcinoma (LUAD), we first performed differential expression analysis on the transcriptomic data from the TCGA-LUAD cohort. A total of 9,404 differentially expressed genes (DEGs) between tumor and adjacent normal tissues were obtained (Fig. 1A and B). By intersecting these DEGs with 84 known PIM pathway genes, we narrowed the focus to 36 candidate genes whose expression levels were altered in LUAD (Fig. 1C, Table S4). Functional enrichment analysis revealed that these genes were predominantly mapped to classical pathways such as phospholipid metabolic processes, regulation of phosphatase activity, and inositol phosphate metabolism (Fig. 1D, Tables S5 and S6), which was consistent with their biological classification. Protein–protein interaction (PPI) network analysis further showed that the proteins encoded by 26 of these 36 genes exhibited interactions, among which PIK3R1, PIK3R2, and PIK3R3 occupied central positions in the network (Fig. 1E), suggesting that they may contribute to PIM pathway-related alterations in LUAD.

### Risk model constructed based on 4 prognostic genes (MTMR7, GDPD1, MTMR4, and MTMR10) demonstrated favorable predictive performance

**Fig. 2:**
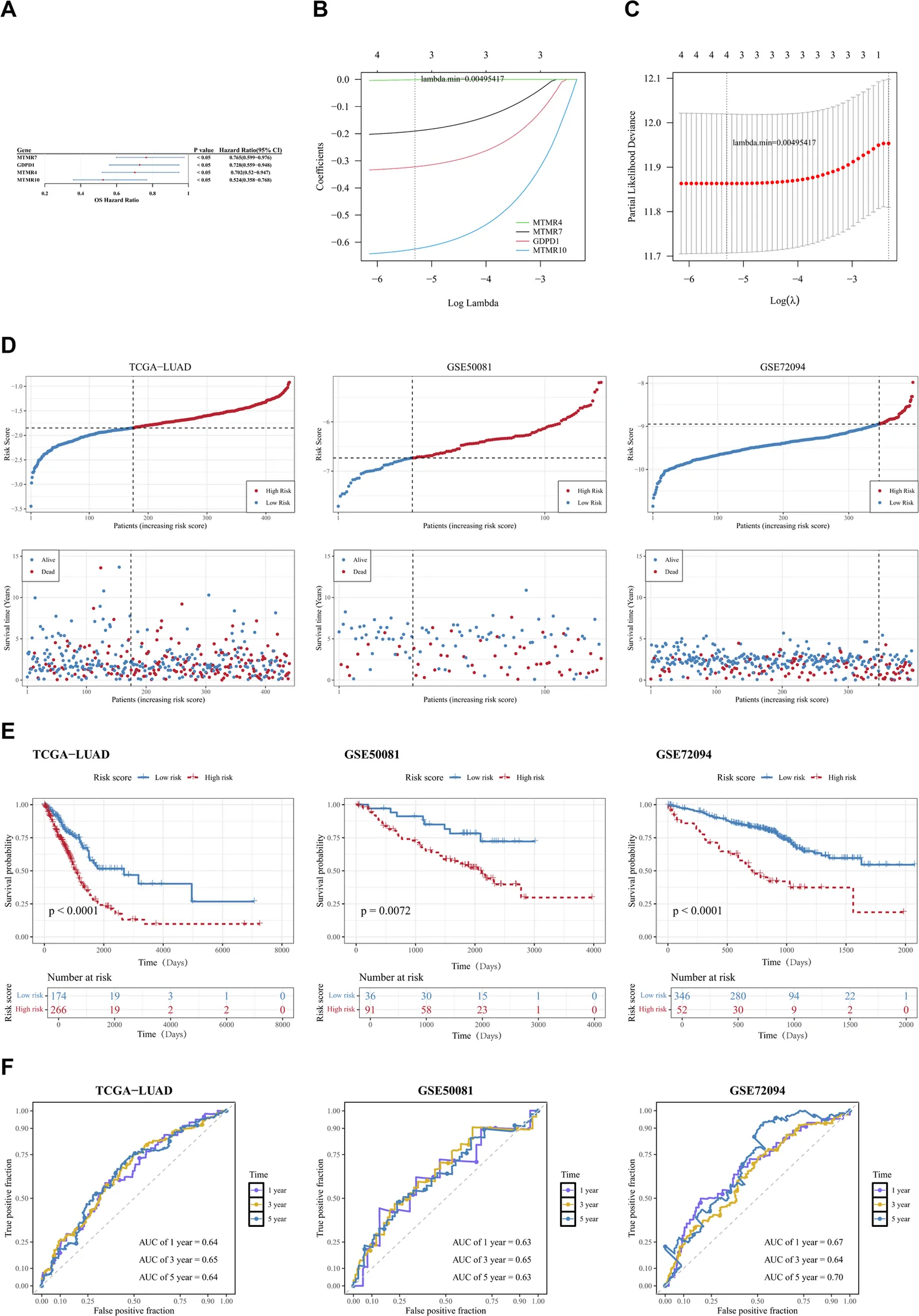
The construction and validation of the risk model. (**A**) The forest plot of candidate prognostic genes screened by univariate Cox regression analysis; (**B**) and (**C**) Regression coefficient path plot and cross-validation curve generated by LASSO model for screening prognostic genes; (**D**) Scatter plots of survival status shown in TCGA-LUAD, GSE50081, and GSE72094; (**E**) K-M curves of TCGA-LUAD, GSE50081, and GSE72094; (**F**) ROC of 1-, 3-, 5-year in TCGA-LUAD, GSE50081, and GSE72094.

Among the 36 candidate genes, we further sought to identify molecules that were statistically associated with overall survival (OS) in LUAD patients. In the TCGA-LUAD, these 36 candidate genes were subjected to univariate Cox regression analysis (*p* < 0.05, HR ≠ 1), followed by PH assumption test (*p* > 0.05), ultimately identifying 4 candidate prognostic genes (MTMR7, GDPD1, MTMR4, and MTMR10) as significantly associated with OS, as depicted in Figs. 2A and S1. Specifically, these 4 genes were all identified as protective factors for LUAD (HR < 1). Subsequently, the 4 genes were subjected to LASSO regression analysis. When the lambda value was 0.00495417, the model was optimal and 4 candidate prognostic genes were all obtained (Fig. 2B and C). These 4 genes, namely MTMR7, GDPD1, MTMR4, and MTMR10, were thus identified as prognostic genes for LUAD.

Based on the expression levels and regression coefficients of these four genes, we established a risk model. The risk score = MTMR7 * (− 0.19) + GDPD1 * (− 0.32) + MTMR4 * (− 0.0009) + MTMR10 * (− 0.63). Thereafter, to evaluate the stability of this score across different datasets, we tested it in the training cohort (TCGA) and two independent validation cohorts (GSE50081 and GSE72094). Based on the optimal risk score cutoff value for each cohort, LUAD patients in each dataset were stratified into a high-risk group (HRG) and a low-risk group (LRG): in the TCGA cohort (cutoff = − 1.849354), there were 266 HRG and 174 LRG cases; in the GSE50081 cohort (cutoff = − 6.733262), there were 91 HRG and 36 LRG cases; and in the GSE72094 cohort (cutoff = − 8.949702), there were 52 HRG and 346 LRG cases. In the 3 datasets, with the increase in risk scores, the probability of patient death all showed an increasing trend (Fig. 2D). Furthermore, the Kaplan–Meier (K-M) survival curve demonstrated that there was a significant difference between the 2 groups, with the HRG showing lower survival odds (TCGA-LUAD: *p* < 0.0001, GSE50081: *p* = 0.0072, GSE72094: *p* < 0.0001) (Fig. 2E). Subsequently, in the aforementioned 3 datasets, the risk model exhibited favorable predictive performance (AUC > 0.6) (Fig. 2F). The above results demonstrated the favorable predictability and robustness of the risk model constructed based on the 4 prognostic genes (MTMR7, GDPD1, MTMR4, and MTMR10).

### Formation of a reliable nomogram model based on independent prognostic factors

**Fig. 3:**
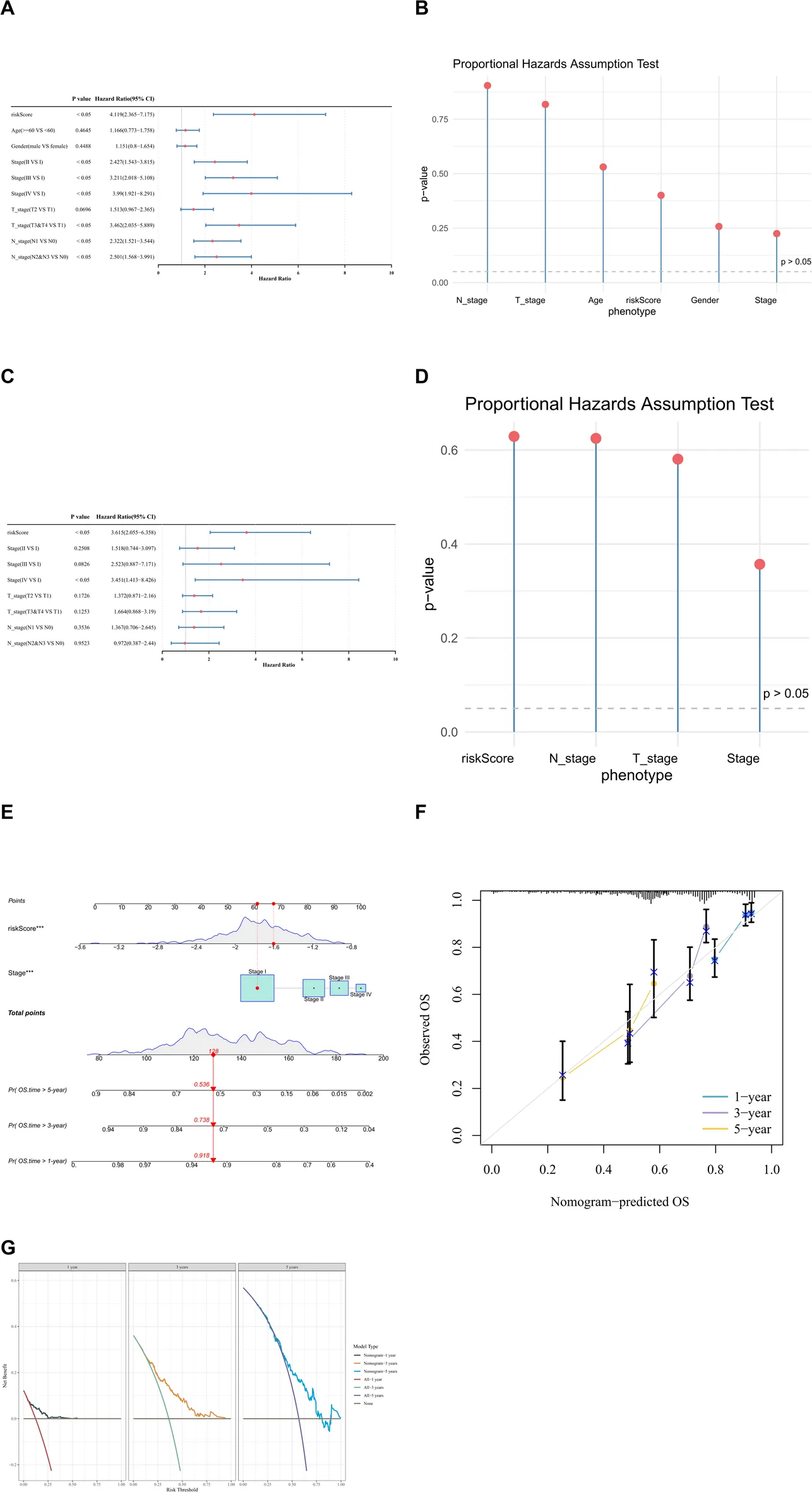
Formation of a reliable nomogram model based on independent prognostic factors. (**A**)–(**D**) Independent prognostic factors screened by univariate and multivariate Cox regression analyses and PH assumption test; (**E**) Nomogram constructed based on risk score and Stage; (**F**) 1-, 3-, 5-year calibration curves of the nomogram; (**G**) 1-, 3-, 5-year DCA curves of the nomogram.

To identify independent prognostic factors, univariate and multivariate Cox regression analyses along with the proportional hazards (PH) assumption test were performed, and both the risk score and tumor stage (stage IV vs. stage I) were confirmed as independent prognostic factors for LUAD (Fig. 3A–D). Based on this result, we integrated these two variables to construct a visualized nomogram model. According to the nomogram, when a patient’s total score for risk score and stage reached 128 points, the model-predicted 1-year, 3-year, and 5-year survival probabilities were 91.8%, 73.8%, and 53.6%, respectively (Fig. 3E). Calibration curves and decision curve analysis (DCA) indicated that the nomogram exhibited satisfactory calibration in predicting survival probability and demonstrated potential clinical net benefit within a certain range of risk thresholds (Fig. 3F and G). These findings suggested that LUAD patients with high risk scores and stage IV tumors tended to have unfavorable prognoses and shorter overall survival.

### Differences in enrichment pathways between HRG and LRG

**Fig. 4:**
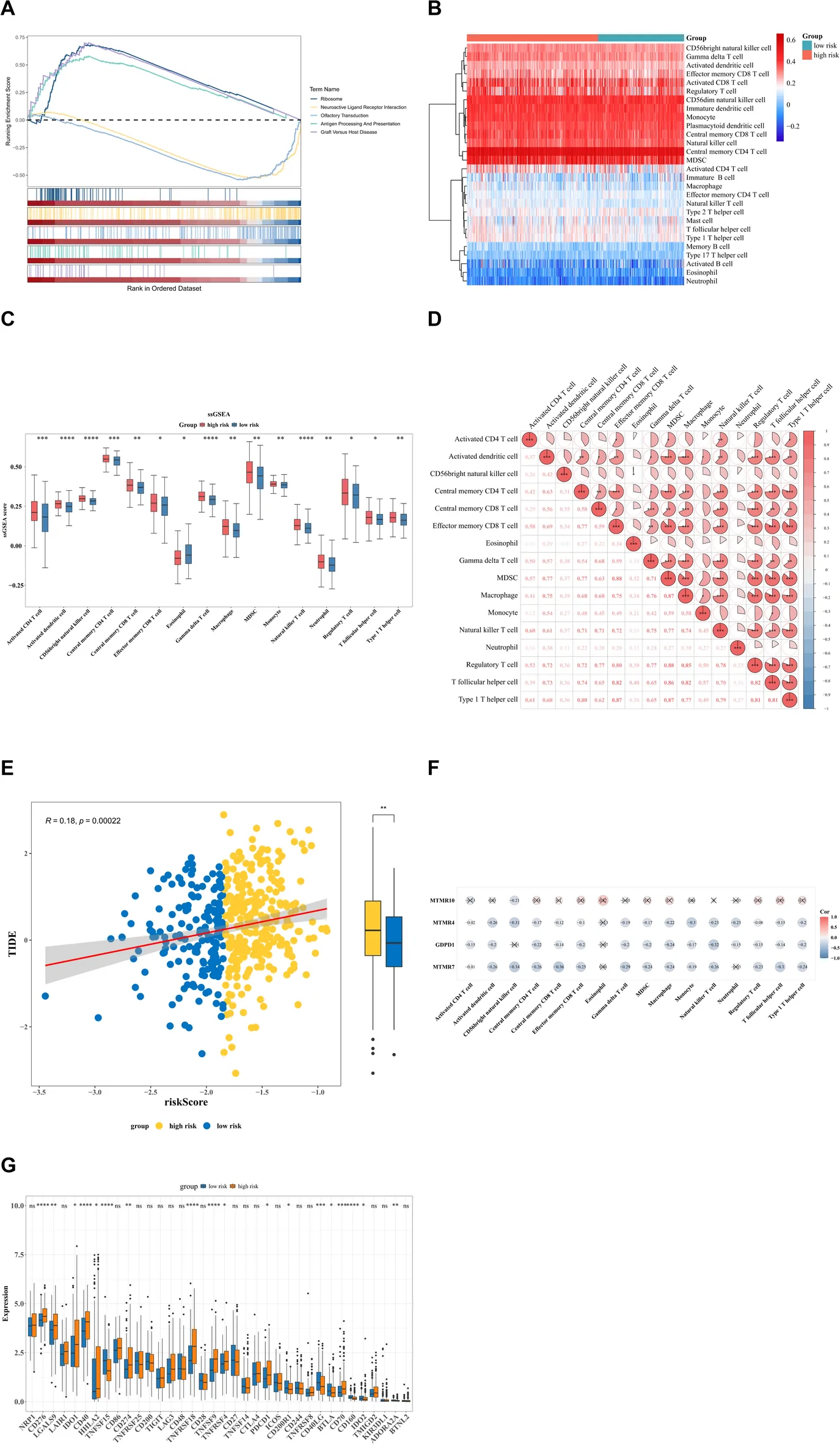
Functional enrichment and tumor microenvironment analyses. (**A**) GSEA of HRG and LRG; (**B**) Immune cell infiltration heatmap of HRG and LRG; (**C**) Boxplot of 16 differentially infiltrated immune cells; (**D**) Correlation heatmap of differentially infiltrated immune cells; (**E**) Boxplot for differential analysis of TIDE scores in HRG and LRG, and scatter plot for correlation between the TIDE score and risk score; (**F**) Correlation heatmap of prognostic genes and differentially infiltrated immune cells; (**G**) Boxplot of the expression of 36 ICGs in the HRG and LRG.

To explore the differences in biological pathways between the high-risk and low-risk groups, the biological pathways involved in HRG and LRG were subjected to further investigation. GSEA revealed that 52 signaling pathways were significantly enriched in these groups (|NES|> 1, FDR < 0.25, *p* < 0.05) (Table S7), the 5 most significant pathways were “ribosome”, “neuroactive ligand receptor interaction”, “olfactory transduction”, “antigen processing and presentation” and “graft versus host disease” (Fig. 4A). The differential enrichment of these pathways indicated that the HRG of LUAD might enhance malignant phenotypes and lead to poor prognosis through multiple mechanisms: enhancing cellular protein synthesis (to support malignant proliferation), abnormally activating neuroendocrine interaction signals (to promote microenvironmental adaptation), regulating ectopic olfactory receptor signals (a potential invasive mechanism), and altering anti-tumor immune responses (associated with immune exhaustion).

### Comprehensive analysis of the tumor microenvironment in LUAD

The immune microenvironment between the HRG and LRG was subjected to further analysis. Figure 4B visualized the immune infiltration profiles of 28 immune cell types in HRG and LRG from TCGA-LUAD. Among them, central memory CD4 T cells and myeloid derived suppressor cells (MDSCs) had relatively high abundance in both HRG and LRG. Marked disparities in infiltration levels of 16 immune cell types were noted between the HRG and LRG (*p* < 0.05), such as activated dendritic cells (*p* = 2.39 × 10^–5^) and gamma delta T cells (*p* = 7.03 × 10^–7^) (Fig. 4C, Table S8). Subsequently, analyses were performed on the correlations both among differentially infiltrated immune cells and between prognostic genes and differentially infiltrated immune cells. The results showed that there was a general positive correlation among differentially infiltrated immune cells. For example, a strong positive correlation was observed between MDSCs and regulatory T cells, as well as between MDSCs and effector memory CD8 T cells (both cor = 0.88, *p* < 0.001) (Fig. 4D). Furthermore, no significant correlations were observed between MTMR10 and differentially infiltrated immune cells. MTMR4 exhibited negative correlations with CD56 bright natural killer (NK) cells (cor = − 0.31) and monocytes (cor = − 0.30). GDPD1 showed a negative correlation only with NK T cells (cor = − 0.32), while MTMR7 displayed significant negative correlations with CD56 bright NK cells (cor = − 0.34), central memory CD8 T cells (cor = − 0.36), and T follicular helper cells (cor = − 0.30) (Fig. 4F). Moreover, the TIDE score of the HRG was higher than that of the LRG (*p* < 0.01). Additionally, the TIDE score was positively correlated with the risk score (Fig. 4E), indicating that a higher risk score was associated with a poorer response of patients to immunotherapy. Further analysis revealed that the expression levels of 18 ICGs differed significantly between the HRG and LRG, with examples including CD276 (*p* = 2.29 × 10^–6^) and TNFSF15 (*p* = 6.228 × 10^–6^) (Fig. 4G, Table S9). These results contributed to the in-depth exploration of the tumor microenvironment in LUAD, thereby laying a foundation for understanding the disease mechanisms and formulating targeted intervention strategies.

### Somatic mutation and chemotherapy sensitivity analyses of LUAD

**Fig. 5:**
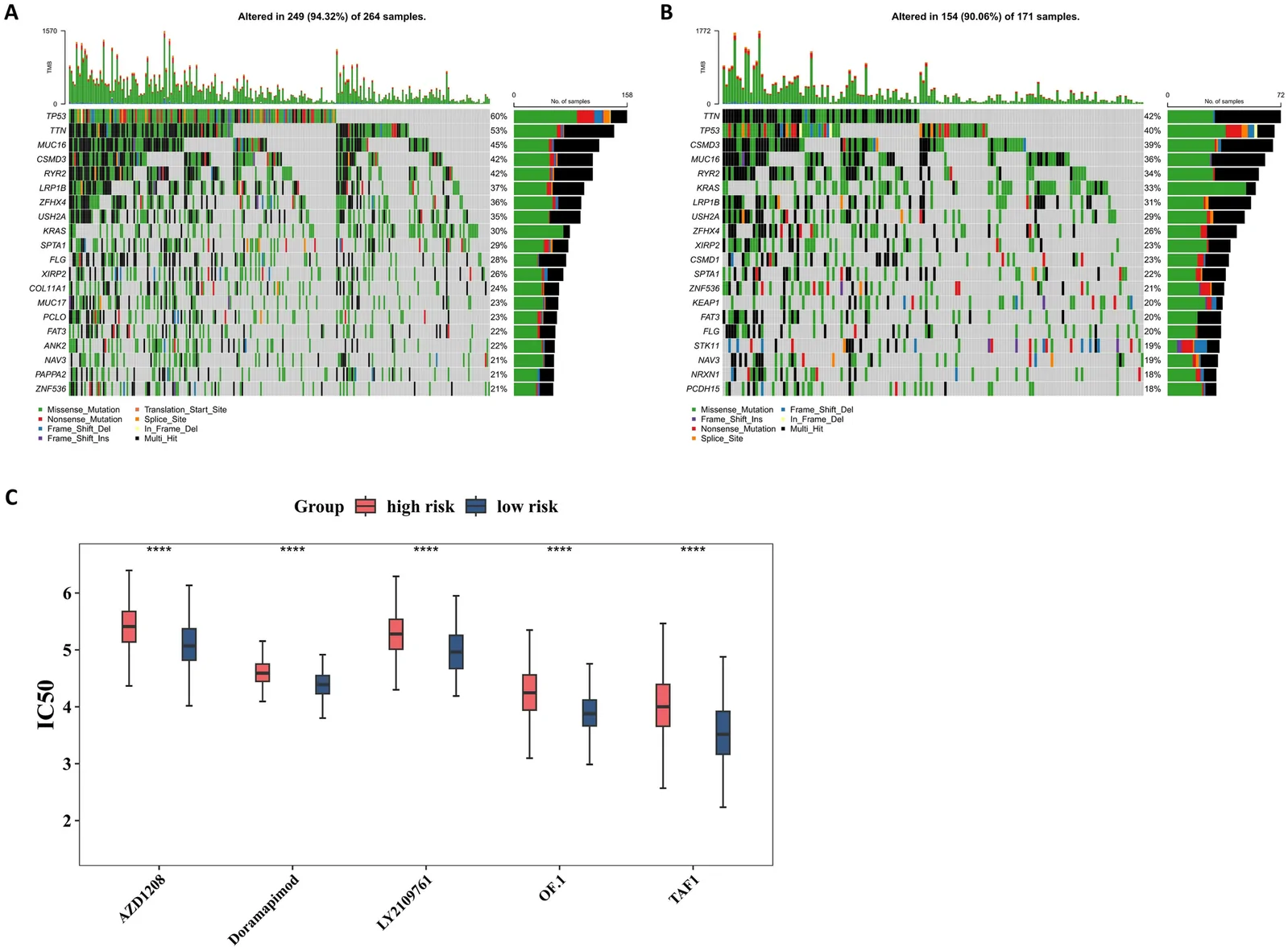
Somatic mutation and chemotherapy sensitivity analyses. (**A**) and (**B**) Waterfall plots of gene mutation profiles in the HRG and LRG; (**C**) Boxplot of differences in drug sensitivity between the HRG and LRG.

Given the critical role of gene mutations in tumor initiation and progression, we analyzed the somatic mutation data of samples from both groups in the TCGA cohort. In the high-risk group, the three genes with the highest mutation frequencies were TP53 (60%), TTN (53%), and MUC16 (45%), whereas in the low-risk group, the three most frequently mutated genes were TTN (42%), TP53 (40%), and CSMD3 (39%) (Fig. 5A and B). Notably, the mutation frequency of TP53 was considerably higher in the high-risk group than in the low-risk group. Further, an analytical investigation was conducted to ascertain the disparities in IC50 levels of chemotherapeutic drugs between the HRG and LRG. It was identified that the IC50 values of 142 chemotherapeutic drugs showed significant differences between HRG and LRG (*p* < 0.05) (Table S10). The top 5 most significantly differential drugs were identified as AZD1208, Doramapimod, LY2109761, OF.1, and TAF1. Additionally, the IC50 values of these drugs in the HRG were all higher than those in the LRG (Fig. 5C), which indicated that high-risk LUAD patients might have lower sensitivity to the aforementioned drugs, suggesting that clinical treatment regimens should be adjusted for this subgroup to improve therapeutic efficacy. The above results of this study provided a theoretical basis and practical reference for exploring potential prognostic risk stratification markers and optimizing the therapeutic strategies for LUAD.

### Epithelial cells were identified as the key cell type

To determine the cell type in which the four prognostic genes were predominantly expressed, we analyzed the single-cell RNA sequencing dataset GSE131907.

**Fig. 6:**
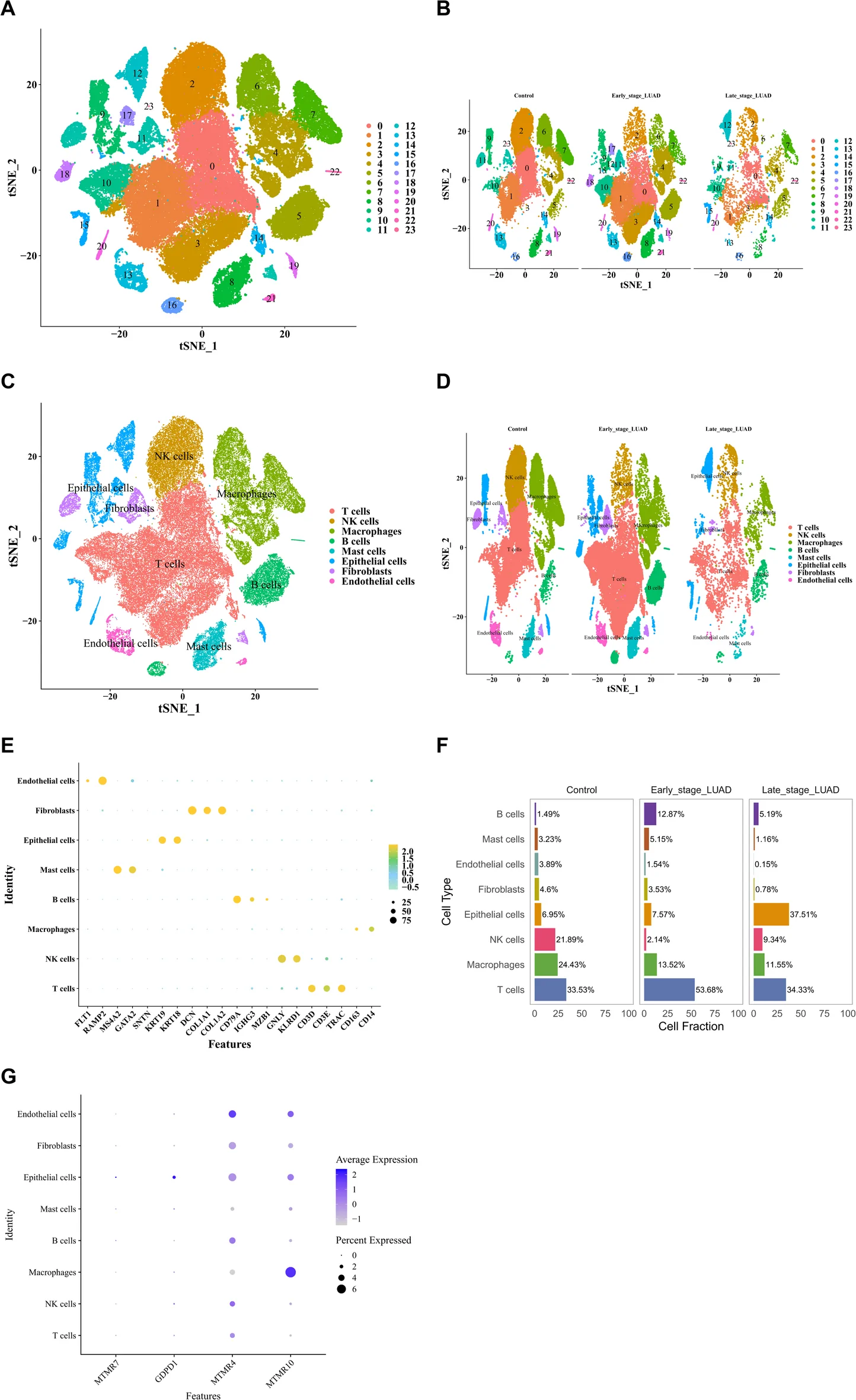
Epithelial cells were identified as the key cell type. (**A**) and (**B**) t-SNE of 24 clusters; (**C**) and (**D**) Cell annotation by t-SNE; (**E**) Expression of marker genes in differently annotated cells; (**F**) Distribution proportions of differently annotated cells; (**G**) Expression of prognostic genes in differently annotated cells.

Following the implementation of QC procedures for the GSE131907, the cell count was reduced from 100,217 to 75,018, while the gene count remained constant at 25,942 (Fig. S2A and B). Next, 2000 HVGs were identified, and the top 10 HVGs were annotated (Fig. S2C). The PCA was performed, and the top 30 PCs were selected for further analysis (*p* < 0.05) (Fig. S2D–E). Thereafter, the 24 clusters were visualized utilizing the t-SNE method (resolution = 0.4) (Fig. 6A and B). The employment of marker genes facilitated the identification of 8 distinct cell types: endothelial cells, mast cells, epithelial cells, fibroblasts, B cells, NK cells, T cells, and macrophages (Fig. 6C). It was found that there were significant differences in the distribution of different cell types among the control group, early-stage LUAD group, and late-stage LUAD group. Specifically, the number of T cells was the highest in the early-stage LUAD group but decreased significantly in the late-stage LUAD group; whereas the number of NK cells in the control group was significantly higher than that in the early-stage and late-stage LUAD groups (Fig. 6D). The specific marker genes associated with each distinct annotated cell type were detailed in Fig. 6E and Table 2. Furthermore, the proportion of T cells was relatively high in all 3 groups. In contrast, the proportion of epithelial cells increased sharply in the late-stage LUAD group, reaching 37.51% (Fig. 6F), which suggested that epithelial cells might play an important role in the progression of LUAD. In addition, overall analysis showed that the 4 prognostic genes (MTMR7, GDPD1, MTMR4, and MTMR10) exhibited higher expression levels in epithelial cells than those in other cell types (Fig. 6G); therefore, epithelial cells were selected as the key cells for subsequent analyses in this study.

**Table 2:** Marker genes for cell annotation.

Cluster	Marker gene	Cell type
13/21	FLT1, RAMP2	Endothelial cells
8	MS4A2, GATA2	Mast cells
9/12/15/17/18/19/20	SNTN, KRT19, KRT18	Epithelial cells
11	DCN, COL1A1, COL1A2	Fibroblasts
5/16/22	CD79A、IGHG3, MZB1	B cells
2/23	GNLY, KLRD1	NK cells
0/1/3/10/14	CD3D, CD3E, TRAC	T cells
4/6/7	CD163, CD14	Macrophages

### Comprehensive analysis of the pathological mechanisms of LUAD at the cellular level

**Fig. 7:**
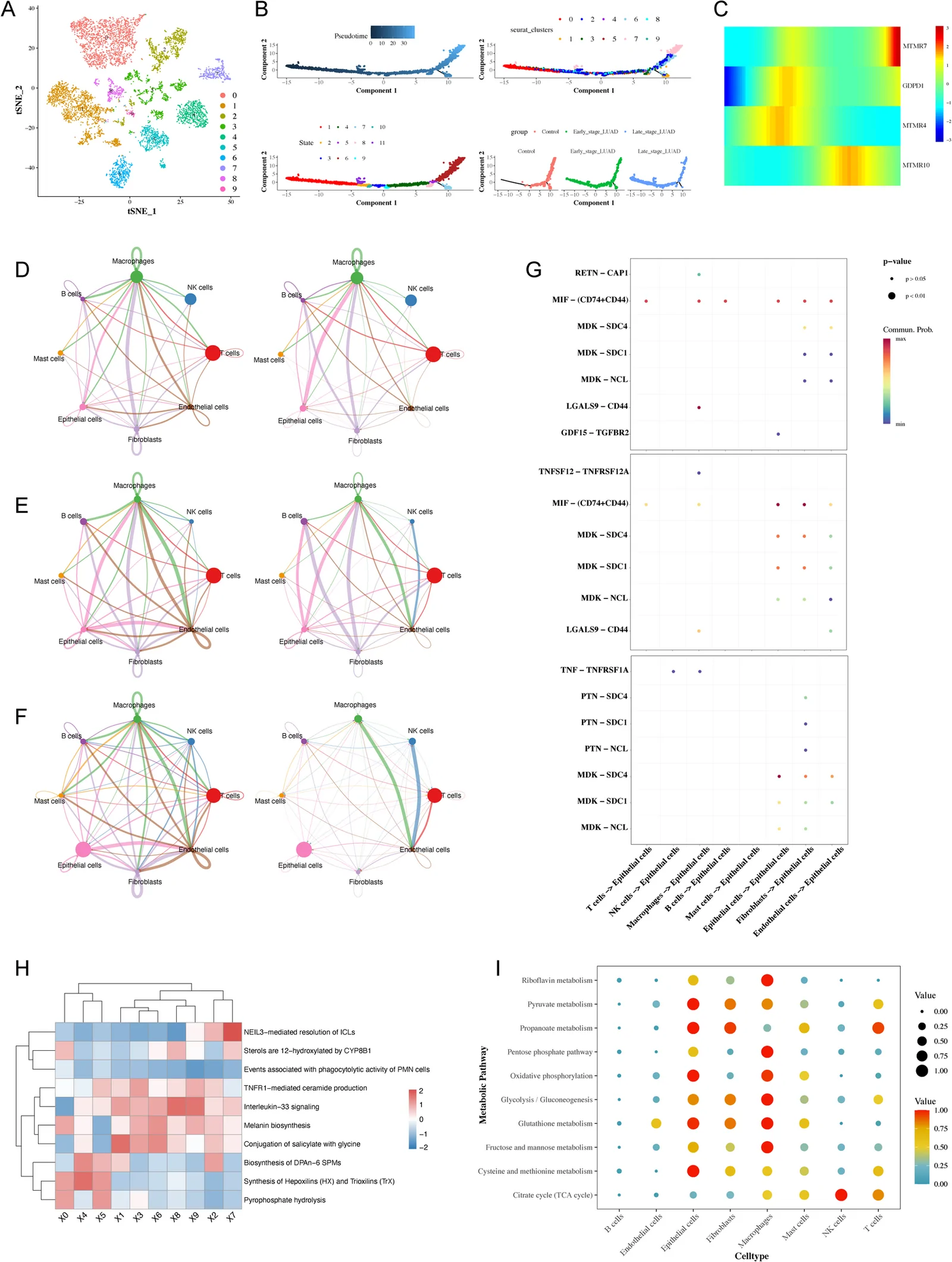
Pseudo-temporal trajectory, cellular communication, enrichment and metabolic analyses. (**A**) t-SNE of 10 clusters of epithelial cells; (**B**) Pseudo-temporal trajectory analysis of epithelial cells; (**C**) Expression changes of prognostic genes observed during Epithelial cells differentiation; (**D**)–(**F**) Cellular communication networks of the control group, early-stage and late-stage LUAD groups; (**G**) Bubble plots of receptor-ligand interaction pairs in the control group, early-stage and late-stage LUAD groups; (**H**) Functional enrichment analysis of epithelial cells; (**I**) Metabolic analysis of epithelial cells.

To further explore the roles of epithelial cells in LUAD, separate clustering analyses of this cell type population were conducted. The results showed that epithelial cells were divided into 10 clusters (resolution = 0.1) (Figs. 7A, S3A and B). Following this, the comprehensive description of the pseudo-temporal trajectory analysis pertaining to epithelial cells was provided. The temporal differentiation of epithelial cells was shown in Fig. 7B. Among these, cluster 0 was primarily formed in the early stage of differentiation, while cluster 2 was distributed throughout almost the entire differentiation process. Over time, epithelial cells differentiated into 11 distinct states. Notably, epithelial cells’ differentiation began at state 1 (the initial state) and concluded at state 6 (the terminal states). In addition, the distribution abundance of epithelial cells in the pseudotime trajectory of the early-stage and late-stage LUAD groups were significantly higher than that in the control group. Next, the dynamic changes in the expression of prognostic genes during epithelial cells differentiation were analyzed. The results showed that MTMR7 exhibited high expression in the late stage of differentiation; GDPD1 and MTMR4 exhibited high expression in the early-to-middle stages of differentiation; and MTMR10 exhibited high expression in the middle-to-late stages of differentiation (Fig. 7C).

Moreover, the examination of cellular communication revealed complex interconnections among the 8 annotated cell types. Compared with the control group, the communication number and weight between epithelial cells and endothelial cells in the early-stage and late-stage LUAD groups were significantly increased. Notably, the communication weight between most cells in the late-stage LUAD group was significantly decreased, but the communication weight between endothelial cells and macrophages, NK cells, and T cells remained at a high level (Fig. 7D–F). Further analysis revealed that in the control group, the communication probability between macrophages and epithelial cells via the LGALS9-CD44 interaction pair was relatively high. For the early-stage LUAD group, fibroblasts and epithelial cells exhibited an elevated likelihood of interacting through the MIF-(CD74 + CD44) interaction pair. In the late-stage LUAD group, by contrast, the interaction between these 2 cell types demonstrated a higher probability of signaling via the MDK-SDC4 interaction pair (Fig. 7G). This finding suggested that the crosstalk among these cells have played an important role in disease progression.

Furthermore, enrichment analysis of epithelial cells yielded a total of 1742 pathways (Table S11). Among these, the top 10 significantly differential pathways encompassed “interleukin-33 signaling”, “events associated with phagocytolytic activity of PMN cells”, and others (Fig. 7H). Moreover, metabolic activity analysis revealed that metabolic pathways such as “cysteine and methionine metabolism”, “glutathione metabolism”, “oxidative phosphorylation”, “propanoate metabolism”, and “pyruvate metabolism” exhibited relatively high activity in epithelial cells (*p* < 0.05) (Fig. 7I). In summary, these results provided a key molecular basis for deciphering the function of epithelial cells in the LUAD microenvironment and the mechanism of LUAD progression, laid a foundation for subsequent LUAD targeted therapy and disease stratification optimization, and further offered new insights for LUAD precision diagnosis and treatment.

### The expression of MTMR7, GDPD1, MTMR4, and MTMR10

**Fig. 8:**
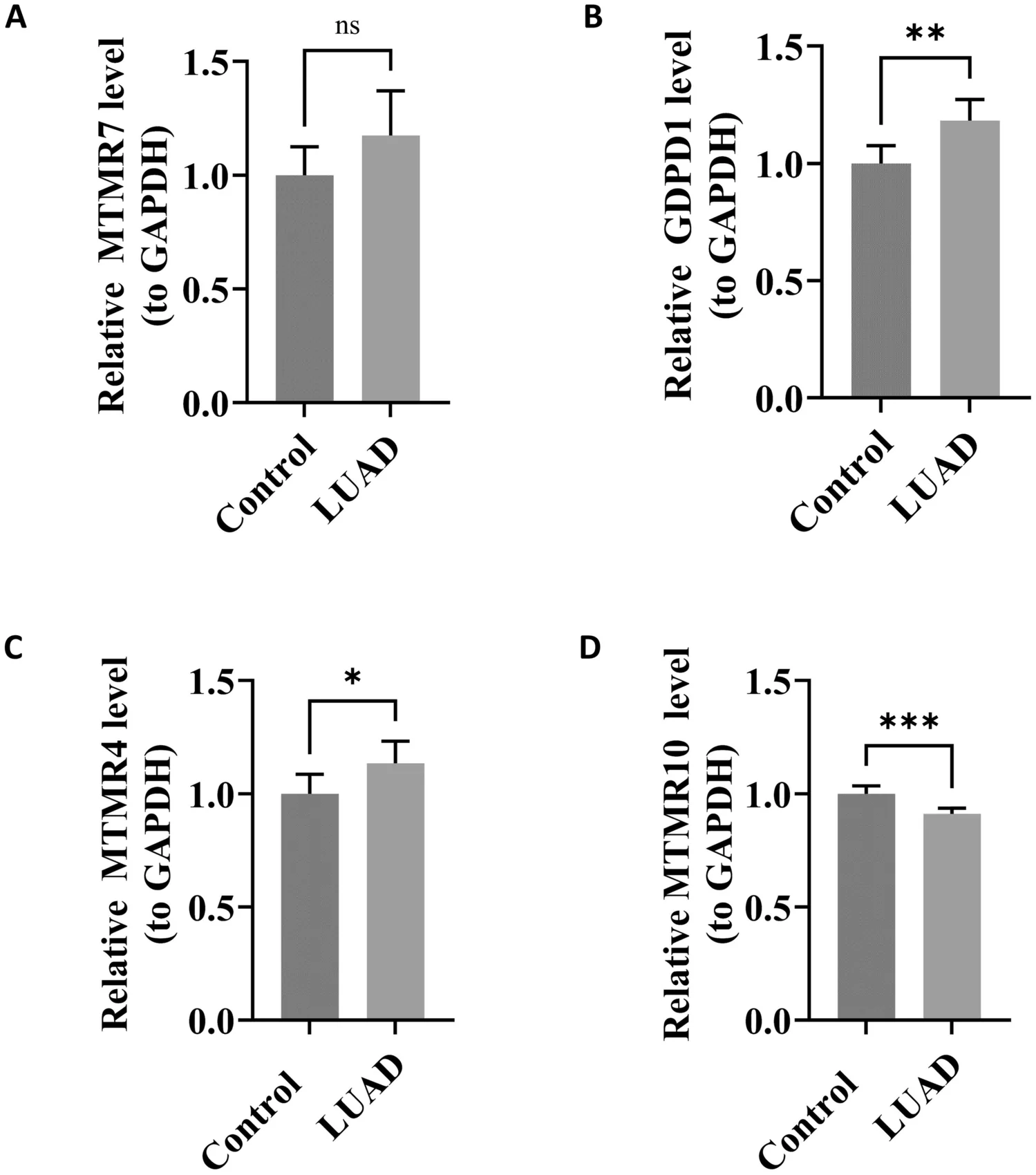
Clinical trial validation of prognostic genes. (**A**)–(**D**) The mRNA expression levels of MTMR7 (**A**), GDPD1 (**B**), MTMR4 (**C**), and MTMR10 (**D**).

Subsequently, the expression levels of 4 prognostic genes (MTMR7, GDPD1, MTMR4, and MTMR10) were investigated in clinical LUAD and control tissue samples via RT-qPCR. The results showed that compared with the control group, the expression levels of GDPD1 and MTMR4 were significantly higher, and the expression level of MTMR10 was significantly downregulated in the LUAD group, which was consistent with the results of the initial differential analysis. Although there was no statistical significance in the expression level of MTMR7 between the two groups, possibly due to the insufficient clinical sample size, its expression level still showed a certain upward trend in the LUAD group (Fig. 8A–D). These findings verified the potential of MTMR7, GDPD1, MTMR4, and MTMR10 as reliable prognostic indicators for LUAD, and laid a foundation for further exploring their roles in the pathogenesis and therapeutic targeting of LUAD.

## Discussion

This study synthesizes bulk and single-cell transcriptomic data to examine the relationship between phosphoinositide metabolism (PIM) and lung adenocarcinoma (LUAD). Through a systematic approach, it identifies four PIM-related genes—MTMR7, GDPD1, MTMR4, and MTMR10—and develops a robust prognostic risk model for LUAD. The single-cell analysis elucidates the dynamic expression patterns of these genes during the differentiation of LUAD epithelial cells. These findings provide valuable molecular insights into the role of phosphoinositide metabolism in the progression of lung adenocarcinoma and offer a promising tool for enhancing prognostic stratification strategies for patients.

The four prognostic genes identified in this study were validated as protective factors for lung adenocarcinoma (LUAD) through univariate Cox regression analysis, indicated by hazard ratios (HR) of less than 1 (Fig. 2A). From an integrative perspective of the PIM pathway, the functions of these genes may not operate independently but rather constitute a potential synergistic protective network. The core dynamic equilibrium of the PIM pathway is regulated through kinase-mediated phosphorylation and phosphatase-mediated dephosphorylation^37^. The downstream PI3K/AKT/mTOR signaling pathway is well-established as being hyperactivated in lung cancer^38^. Within this context, GDPD1, a pivotal enzyme in glycerophospholipid metabolism^39^, may indirectly contribute to the metabolic support of the PIM network by modulating the availability of lipid precursors necessary for phosphatidylinositol synthesis^40,41^. In the context of the MTMR lipid phosphatase family, MTMR7, MTMR4, and MTMR10 play distinct roles in regulating various stages of phosphatidylinositol monophosphate (PIM) dephosphorylation, as evidenced by several studies^42–44^. Specifically, MTMR7 is capable of modulating intracellular signal transduction through the dephosphorylation of specific phosphatidylinositols^42^ and may inhibit PI3K signaling pathways activated by KRAS mutations^45^. MTMR4 is implicated in the regulation of phosphoinositides within the endosomal-lysosomal compartment and is associated with autophagic processes^46,47^. In contrast, MTMR10 functions as a pseudophosphatase, having lost independent catalytic activity^44^, yet it can indirectly influence the PI3P/PI(3,5)P_2_ pool by forming heterocomplexes with catalytically active MTMR members^48^. The RT-qPCR validation results indicated that, in comparison to the control group, the mRNA levels of GDPD1 and MTMR4 were significantly elevated in the LUAD group. Additionally, MTMR7 demonstrated an upward trend, whereas MTMR10 was significantly downregulated. These findings are largely consistent with the differential analysis results obtained from the TCGA database. Interestingly, there exists an apparent paradox between the elevated expression of MTMR4, MTMR7, and GDPD1 in tumor tissues and their roles as protective factors. We hypothesize that this may represent a compensatory phenomenon. The increased expression of these three genes in LUAD may reflect a compensatory activation of cancer cells under conditions of an imbalanced PIM network; however, this compensation ultimately proves insufficient to counteract the persistent oncogenic signaling.

Single-cell analysis has yielded essential insights into the synergistic mechanisms of these four genes. The selection of epithelial cells as the key cell type is supported by their role as the cells of origin in LUAD, including AT2 and club cells^49^. Their increased proportion in late-stage LUAD (Fig. 6F) reflects malignant clonal expansion, while EMT and epithelial plasticity further highlight their role in LUAD progression. Pseudotime trajectory analysis further elucidated that these four genes display distinct temporal expression patterns throughout epithelial cell differentiation: GDPD1 and MTMR4 are predominantly expressed in the early stages of differentiation, MTMR10 exhibits increased expression in the mid-to-late stages, and MTMR7 reaches peak expression during the late stage of differentiation. Based on the correlation between the aforementioned functional characteristics and temporal expression patterns, we propose that these four genes may operate synergistically at various stages of epithelial cell differentiation, collectively establishing a multi-layered protective network against PIM. During the initial phase of differentiation, GDPD1 is responsible for maintaining glycerophospholipid metabolic homeostasis, thereby ensuring substrate availability, while MTMR4 modulates phosphoinositides within endosome-lysosome compartments to preserve membrane trafficking integrity. As differentiation progresses to the mid-to-late phase, MTMR10 contributes indirectly to the precise regulation of the phosphoinositide pool through the formation of heterocomplexes. In the terminal phase of differentiation, MTMR7 continues to uphold membrane trafficking order, thereby preventing the excessive activation of the PI3K/AKT pathway. Should the protective efficacy of these four genes in LUAD be compromised due to imbalanced expression, the multi-layered and multi-stage regulatory mechanisms from early to late differentiation may become systematically disrupted, potentially facilitating the persistent activation of the PI3K/AKT/mTOR pathway and consequently driving tumor progression. The proposed framework synthesizes prognostic associations at the bulk level, expression characteristics at the single-cell level, and expression validation in clinical samples, thereby offering an initial conceptual model for elucidating the synergistic roles of PIM-related genes in lung adenocarcinoma (LUAD). Nonetheless, the precise regulatory mechanisms necessitate further validation through functional experiments (refer to Fig. S4).

Significant differences in biological functions and immune microenvironment characteristics were observed between high-risk and low-risk groups, offering further potential insights into the influence of PIM-related genes on the progression of lung adenocarcinoma (LUAD). Gene Set Enrichment Analysis (GSEA) indicated that the high-risk group exhibited significant enrichment in pathways such as the ribosome pathway, neuroactive ligand-receptor interaction, and antigen processing and presentation. Notably, previous research has demonstrated that MTMR7 and MTMR10 are expressed in brain tissue and are implicated in neural development and stress responses^50–53^. Furthermore, the α7-nAChR subtype within the neuroactive ligand-receptor interaction pathway has been identified as a crucial cancer-promoting mediator in lung cancer^54^. This background indicates that the differential enrichment observed between high-risk and low-risk groups within this pathway may be associated with alterations in the expression of MTMR7 and MTMR10. Both genes could play an indirect role in remodeling the tumor microenvironment of lung adenocarcinoma (LUAD) by influencing neuroactive ligand-related signal transduction. Furthermore, the aberrant activation of the ribosome pathway implies that tumor cells within the high-risk group may exhibit an enhanced capacity for protein synthesis, thereby facilitating their malignant proliferation. Prior research has established that increased ribosome biogenesis contributes to acquired resistance to osimertinib in LUAD cells^55^, while diminished ribosomal activity may improve the effectiveness of immunotherapy^56^. Considering the regulatory function of the PIM downstream PI3K/AKT/mTOR pathway in ribosome biogenesis^57^, the observed differences in enrichment suggest that an imbalance in PIM signaling within the high-risk group may lead to excessive activation of the ribosome pathway via an mTOR-dependent mechanism, thereby synergistically promoting tumor proliferation and treatment resistance.

The analysis of the immune microenvironment further elucidated distinct variations in anti-tumor immune characteristics between the two groups. Notably, significant differences were observed in the infiltration levels of 16 distinct immune cell types between the high-risk and low-risk cohorts. Additionally, the TIDE score was markedly elevated in the high-risk group, indicating an increased potential for immune evasion. Correlation analysis revealed that MTMR7 exhibited a negative correlation with central memory CD8⁺ T cells and follicular helper T cells, MTMR4 was negatively correlated with CD56 bright NK cells and monocytes, and GDPD1 was negatively correlated with NKT cells. This selective pattern of negative correlation implies that the aforementioned PIM-related genes may modulate local immune responses in lung adenocarcinoma (LUAD) by influencing the infiltration or functional status of specific immune cell subsets. Previous studies have demonstrated that the enrichment of follicular helper T cells can potentiate the anti-tumor immune response of CD8⁺ T cells and is associated with improved survival outcomes in LUAD patients^58,59^. In this study, a negative correlation was observed between MTMR7 and this particular cell subset, suggesting its potential immunomodulatory function within the tumor microenvironment to mitigate tissue damage resulting from excessive immune responses. Nevertheless, this function may be compromised in the presence of a broader imbalance within the PIM network, which could facilitate immune evasion. Future research should focus on conducting more comprehensive investigations in this immune-related context.

In recent years, there has been an increasing number of studies dedicated to the development of prognostic models for lung adenocarcinoma (LUAD) utilizing transcriptomic data. For instance, Hua et al. constructed a prognostic signature comprising seven genes associated with immune checkpoint pathways, employing the TCGA and GSE72094 datasets, and demonstrated promising outcomes in predicting responses to immunotherapy^60^. Although this study shares some data sources with the aforementioned research, it diverges in its research focus and analytical dimensions. This study delves into the biological theme of phosphoinositide metabolism, a prognostic aspect of LUAD that has yet to be systematically investigated. The four identified genes are implicated in distinct regulatory nodes within the PIM network, thereby providing a metabolic perspective that enhances the immune checkpoint viewpoint in assessing LUAD prognosis. Notably, a gene-by-gene comparison of ICG expression patterns between the two studies (Table S12) reveals both convergence and distinction: BTLA and CD200R1 showed concordant patterns across both stratification systems, whereas CD274 and TNFSF18 exhibited directional discordance attributable to the distinct biological bases of the two models (ICG-driven vs. PIM-driven); moreover, several ICGs not highlighted in Hua et al.—including CD276, IDO1, and TNFSF15—were significantly differentially expressed in the present study, suggesting that PIM dysregulation engages immune checkpoint axes beyond the classical PD-1/PD-L1 pathway. From an analytical standpoint, this research incorporates single-cell RNA sequencing analysis alongside bulk transcriptomics, uncovering the temporal expression patterns of the four PIM-related genes during epithelial cell differentiation. This dynamic, cellular-level insight adds an additional layer of understanding regarding the role of the PIM network in LUAD progression. In conclusion, by integrating multi-omics data within the framework of PIM, this study presents a novel and complementary perspective for molecular subtyping and prognostic evaluation in LUAD.

However, this study is subject to several limitations, including a restricted sample size within the database, inadequate details regarding the samples, and a need for more comprehensive evidence-based medical validation to substantiate the clinical applicability of the model. Additionally, the risk subgroups (HRG/LRG) established in this study using TCGA bulk transcriptomic data and the early/late subgroups identified in the single-cell data originate from different cohorts. Furthermore, discrepancies in sequencing platforms and batch processing methods hinder the direct mapping of single-cell samples to the bulk-derived risk classification system. Consequently, this study does not directly elucidate the correspondence between the two classification frameworks. Furthermore, the interaction genes and associated biological pathways identified herein necessitate further validation to establish robust evidence for clinical targeted therapy. While this research provides novel insights from a bioinformatics perspective into the relationship between LUAD and PIM, future investigations should utilize larger-scale clinical cohorts incorporating paired bulk and single-cell data, alongside gene function studies and animal model research, to substantiate the reliability and biological significance of the current findings. Meanwhile, we will continue to monitor advancements in research concerning the aforementioned prognostic genes in LUAD, with the aim of enhancing the understanding of their underlying functional mechanisms.

## Conclusion

To sum up, our research has discovered a new 4-gene signature that independently predicts outcomes in lung adenocarcinoma patients and may also serve as potential therapeutic targets.

## Supplementary Information

Supplementary Information 1.

Supplementary Information 2.

Supplementary Information 3.

Supplementary Information 4.

Supplementary Information 5.

Supplementary Information 6.

Supplementary Information 7.

Supplementary Information 8.

Supplementary Information 9.

Supplementary Information 10.

Supplementary Information 11.

Supplementary Information 12.

Supplementary Information 13.

Supplementary Information 14.

Supplementary Information 15.

Supplementary Information 16.

## Data Availability

All raw data and code are available upon request.

## Abbreviations

LUAD: Lung adenocarcinoma
NSCLC: Non-small cell lung cancer
PIM: Phosphoinositides metabolism
PIM-RGs: PIM-related genes
DEGs: Differentially expressed genes
GO: Gene ontology
KEGG: Kyoto Encyclopedia of Genes and Genomes
LASSO: Least absolute shrinkage and selection operator
PH: Proportional hazards
HRG: High-risk group
LRG: Low-risk group
ROC: Receiver operating characteristic
AUC: Area under curve
GSEA: Gene set enrichment analysis
TIDE: Tumor Immune Dysfunction and Exclusion
RT-qPCR: Reverse transcription quantitative PCR
scRNA-seq: Single-cell RNA sequencing
TCGA: The Cancer Genome Atlas
GEO: Gene Expression Omnibus
MSigDB: The Molecular Signatures Database
STRING: Search Tool for the Retrieval of Interacting Genes
PPI: Protein-protein interaction
OS: Overall survival
BP: Biological process
CC: Cellular component
MF: Molecular function
ICGs: Immune checkpoint genes
K-M: Kaplan-Meier
DCA: Decision curve analysis
NES: Normalized enrichment score
FDR: False discovery rate
GDSC: Drug Sensitivity in Cancer
IC50: Half-maximal inhibitory concentration
HVGs: Highly variable genes
VST: Variance-stabilizing transformation
PCA: Principal component analysis
PCs: Principal components
t-SNE: T-distributed stochastic neighbor embedding
