# Supplementary material for: Bulk and single-cell transcriptomics reveal prognostic signatures of phosphoinositide metabolism in lung adenocarcinoma
Source: Sci Rep. 2026 Jun 10;16:26449. doi: 10.1038/s41598-026-56684-2 (PMC13500466; doi:10.1038/s41598-026-56684-2)
Supplement: Supplementary file 1 [file 41598_2026_56684_MOESM1_ESM.pdf]

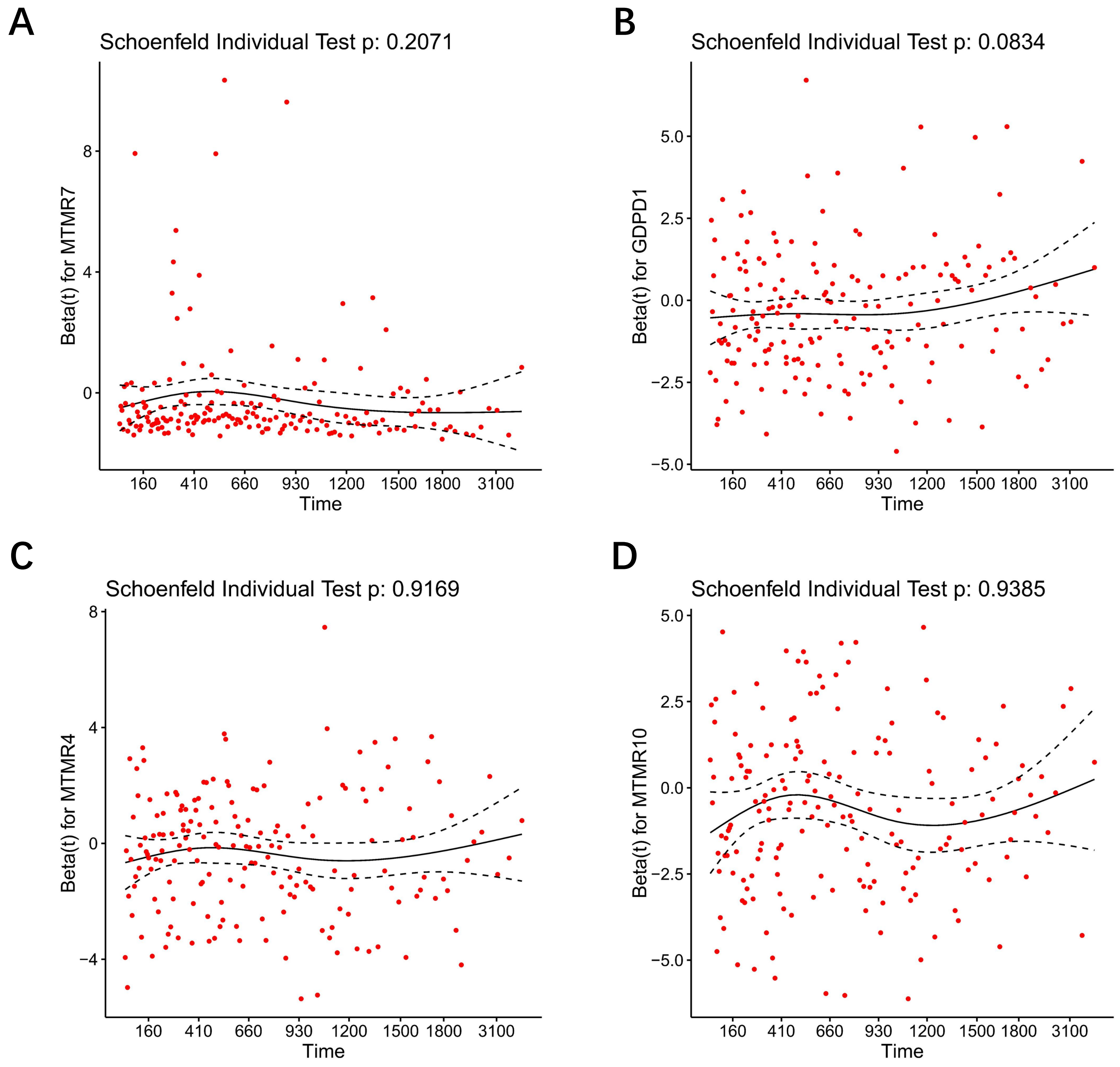
A
Schoenfeld Individual Test p: 0.2071
Beta(t) for MTMR7
Time
B
Schoenfeld Individual Test p: 0.0834
Beta(t) for GDPD1
Time
C
Schoenfeld Individual Test p: 0.9169
Beta(t) for MTMR4
Time
D
Schoenfeld Individual Test p: 0.9385
Beta(t) for MTMR10
Time
160 410 660 930 1200 1500 1800 3100
