# Supplementary material for: Bulk and single-cell transcriptomics reveal prognostic signatures of phosphoinositide metabolism in lung adenocarcinoma
Source: Sci Rep. 2026 Jun 10;16:26449. doi: 10.1038/s41598-026-56684-2 (PMC13500466; doi:10.1038/s41598-026-56684-2)
Supplement: Supplementary file 2 [file 41598_2026_56684_MOESM2_ESM.pdf]

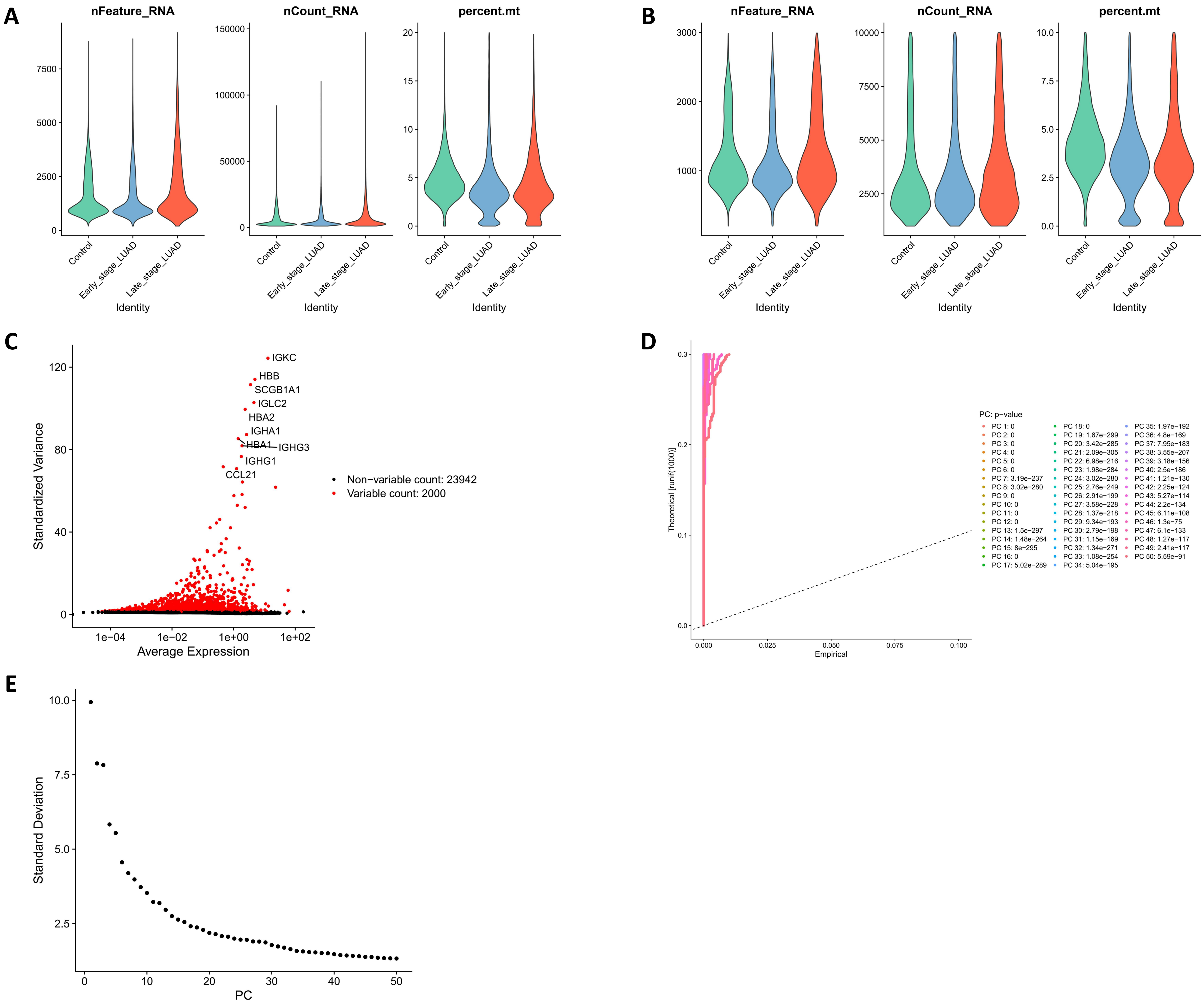
A
nFeature_RNA
nCount_RNA
percent.mt
Control
Early_stage_LUAD
Late_stage_LUAD
Identity
B
nFeature_RNA
nCount_RNA
percent.mt
Identity
C
IGKC
HBB
SCGB1A1
IGLC2
HBA2
IGHA1
HBA1
IGHG3
IGHG1
CCL21
Standardized Variance
Average Expression
Non−variable count: 23942
Variable count: 2000
D
Theoretical [runif(1000)]
Empirical
PC: p−value
PC 1: 0
PC 2: 0
PC 3: 0
PC 4: 0
PC 5: 0
PC 6: 0
PC 7: 3.19e−237
PC 8: 3.02e−280
PC 9: 0
PC 10: 0
PC 11: 0
PC 12: 0
PC 13: 1.5e−297
PC 14: 1.48e−264
PC 15: 8e−295
PC 16: 0
PC 17: 5.02e−289
PC 18: 0
PC 19: 1.67e−299
PC 20: 3.42e−285
PC 21: 2.09e−305
PC 22: 6.98e−216
PC 23: 1.98e−284
PC 24: 3.02e−280
PC 25: 2.76e−249
PC 26: 2.91e−199
PC 27: 3.58e−228
PC 28: 1.37e−218
PC 29: 9.34e−193
PC 30: 2.79e−198
PC 31: 1.15e−169
PC 32: 1.34e−271
PC 33: 1.08e−254
PC 34: 5.04e−195
PC 35: 1.97e−192
PC 36: 4.8e−169
PC 37: 7.95e−183
PC 38: 3.55e−207
PC 39: 3.18e−156
PC 40: 2.5e−186
PC 41: 1.21e−130
PC 42: 2.25e−124
PC 43: 5.27e−114
PC 44: 2.2e−134
PC 45: 6.11e−108
PC 46: 1.3e−75
PC 47: 6.1e−133
PC 48: 1.27e−117
PC 49: 2.41e−117
PC 50: 5.59e−91
E
Standard Deviation
PC
