# Supplementary material for: Bulk and single-cell transcriptomics reveal prognostic signatures of phosphoinositide metabolism in lung adenocarcinoma
Source: Sci Rep. 2026 Jun 10;16:26449. doi: 10.1038/s41598-026-56684-2 (PMC13500466; doi:10.1038/s41598-026-56684-2)
Supplement: Supplementary file 3 [file 41598_2026_56684_MOESM3_ESM.pdf]

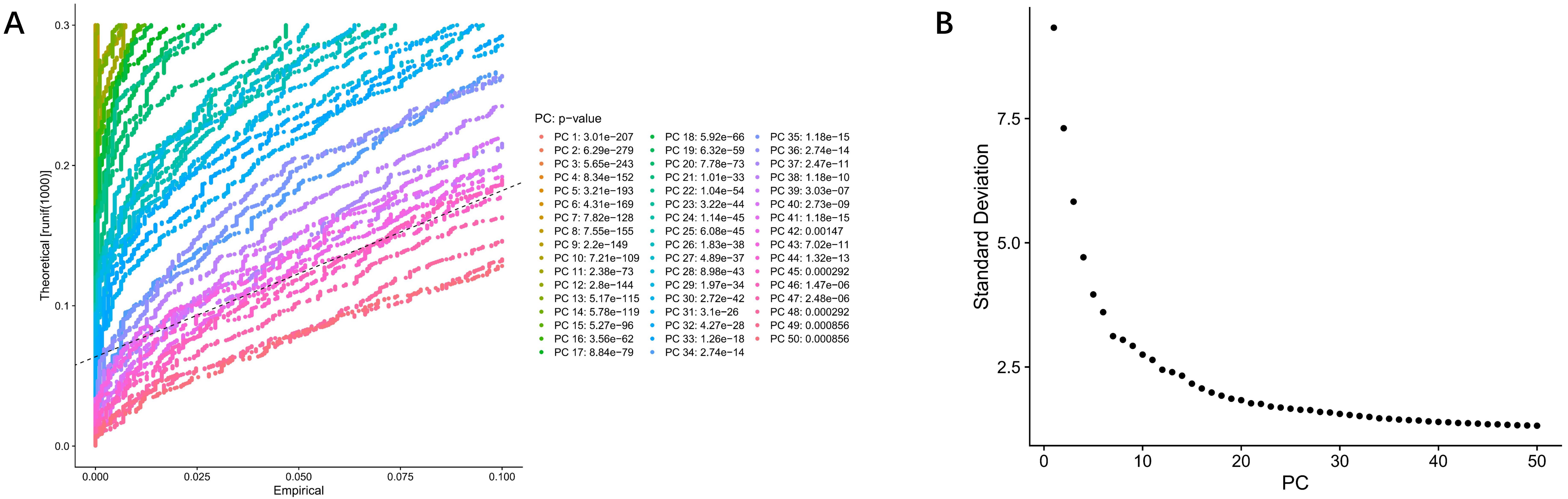
A
Theoretical [runif(1000)]
Empirical
0.0
0.1
0.2
0.3
0.000
0.025
0.050
0.075
0.100
PC: p−value
PC 1: 3.01e−207
PC 2: 6.29e−279
PC 3: 5.65e−243
PC 4: 8.34e−152
PC 5: 3.21e−193
PC 6: 4.31e−169
PC 7: 7.82e−128
PC 8: 7.55e−155
PC 9: 2.2e−149
PC 10: 7.21e−109
PC 11: 2.38e−73
PC 12: 2.8e−144
PC 13: 5.17e−115
PC 14: 5.78e−119
PC 15: 5.27e−96
PC 16: 3.56e−62
PC 17: 8.84e−79
PC 18: 5.92e−66
PC 19: 6.32e−59
PC 20: 7.78e−73
PC 21: 1.01e−33
PC 22: 1.04e−54
PC 23: 3.22e−44
PC 24: 1.14e−45
PC 25: 6.08e−45
PC 26: 1.83e−38
PC 27: 4.89e−37
PC 28: 8.98e−43
PC 29: 1.97e−34
PC 30: 2.72e−42
PC 31: 3.1e−26
PC 32: 4.27e−28
PC 33: 1.26e−18
PC 34: 2.74e−14
PC 35: 1.18e−15
PC 36: 2.74e−14
PC 37: 2.47e−11
PC 38: 1.18e−10
PC 39: 3.03e−07
PC 40: 2.73e−09
PC 41: 1.18e−15
PC 42: 0.00147
PC 43: 7.02e−11
PC 44: 1.32e−13
PC 45: 0.000292
PC 46: 1.47e−06
PC 47: 2.48e−06
PC 48: 0.000292
PC 49: 0.000856
PC 50: 0.000856
B
Standard Deviation
PC
2.5
5.0
7.5
0
10
20
30
40
50
