# Supplementary material for: Bulk and single-cell transcriptomics reveal prognostic signatures of phosphoinositide metabolism in lung adenocarcinoma
Source: Sci Rep. 2026 Jun 10;16:26449. doi: 10.1038/s41598-026-56684-2 (PMC13500466; doi:10.1038/s41598-026-56684-2)
Supplement: Supplementary file 4 [file 41598_2026_56684_MOESM4_ESM.pdf]

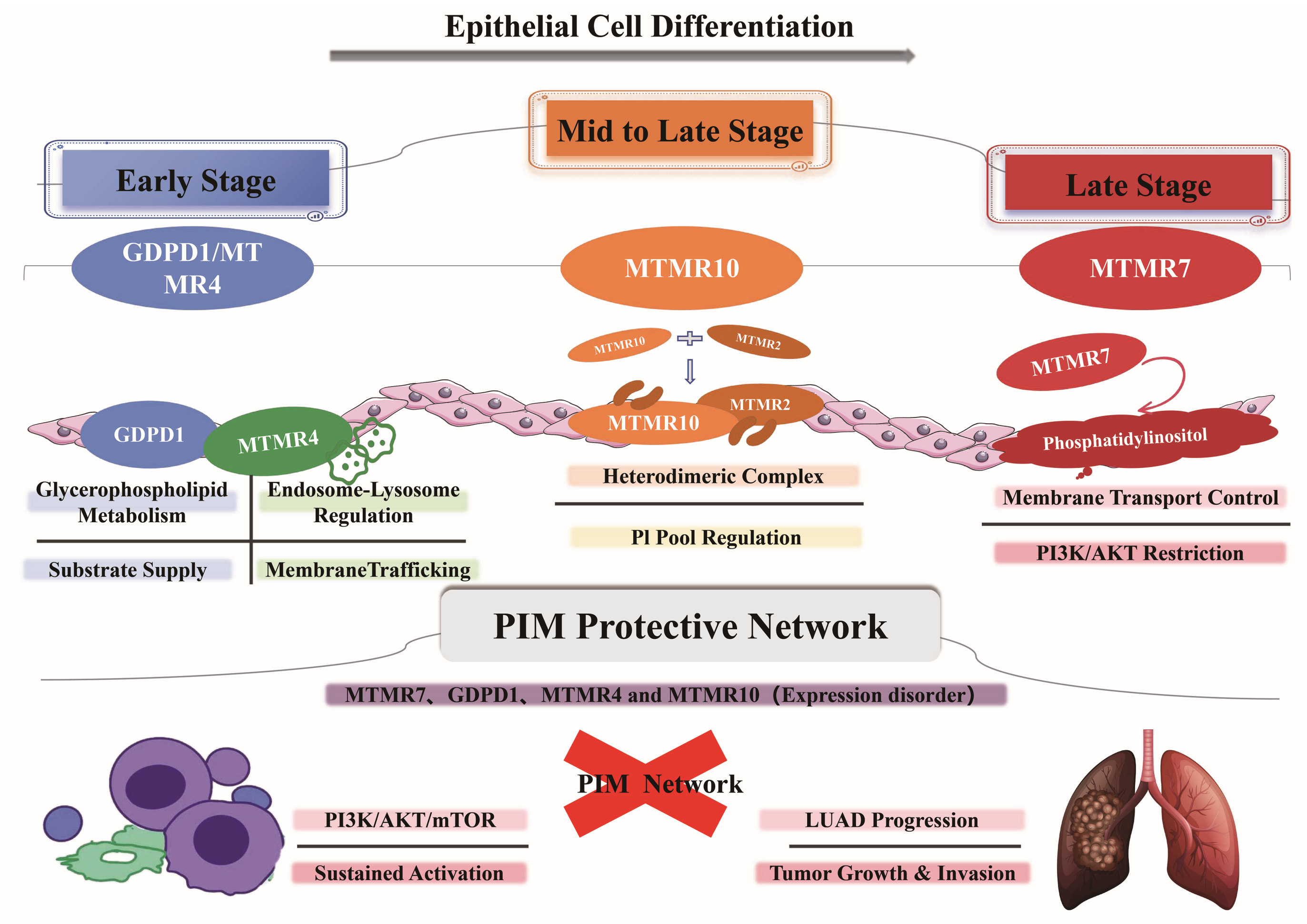
Epithelial Cell Differentiation
Early Stage
Mid to Late Stage
Late Stage
GDPD1/MTMR4
MTMR10
MTMR7
MTMR10
MTMR2
MTMR7
GDPD1
MTMR4
MTMR10
MTMR2
Phosphatidylinositol
Glycerophospholipid Metabolism
Endosome-Lysosome Regulation
Substrate Supply
MembraneTrafficking
Heterodimeric Complex
Pl Pool Regulation
Membrane Transport Control
PI3K/AKT Restriction
PIM Protective Network
MTMR7、GDPD1、MTMR4 and MTMR10（Expression disorder）
PIM Network
PI3K/AKT/mTOR
Sustained Activation
LUAD Progression
Tumor Growth & Invasion
