# Supplementary material for: Bulk and single-cell transcriptomics reveal prognostic signatures of phosphoinositide metabolism in lung adenocarcinoma
Source: Sci Rep. 2026 Jun 10;16:26449. doi: 10.1038/s41598-026-56684-2 (PMC13500466; doi:10.1038/s41598-026-56684-2)
Supplement: Supplementary file 5 [file 41598_2026_56684_MOESM16_ESM.docx]

# Comparison of ICG Expression Patterns: Hua et al. (2023) vs. This Study

*Note: In Hua et al., C2 = better-prognosis cluster; in this study, LRG = low-risk group (better prognosis), HRG = high-risk group. Significance: * p<0.05, ** p<0.01, *** p<0.001, **** p<0.0001; ns = not significant.*

| Gene | Hua et al. expression | Hua et al. significance | This study expression | This study significance | Interpretation |
| --- | --- | --- | --- | --- | --- |
| Concordant — same direction, significant in both studies | | | | | |
| BTLA | Higher in C2 | *** | Higher in LRG | * | Consistent across both models |
| CD200R1 | Higher in C2 | *** | Higher in LRG | * | Consistent across both models |
| Discordant — opposite direction, significant in both studies | | | | | |
| CD274 | Higher in C2 | *** | Higher in HRG | ** | Reflects different model construction basis |
| TNFSF18 | Higher in C2 | *** | Higher in HRG | **** | Reflects different model construction basis |
| Significant in Hua et al. only — not significant in this study | | | | | |
| LAIR1 | Higher in C2 | *** | — | ns | ICG-driven stratification feature |
| CD244 | Higher in C2 | *** | — | ns | ICG-driven stratification feature |
| ICOS | Higher in C2 | *** | — | ns | ICG-driven stratification feature |
| CD48 | Higher in C2 | *** | — | ns | ICG-driven stratification feature |
| CD28 | Higher in C2 | *** | — | ns | ICG-driven stratification feature |
| CD86 | Higher in C2 | *** | — | ns | ICG-driven stratification feature |
| Novel findings — significant in this study only (PIM-specific) | | | | | |
| CD276 | — | Not reported | Higher in HRG | **** | Inhibitory checkpoint; associated with immune evasion |
| IDO1 | — | Not reported | Higher in HRG | * | Tryptophan depletion; immunosuppression |
| CD40 | — | Not reported | Higher in HRG | **** | Tumor-promoting context in solid tumors |
| TNFSF9 | — | Not reported | Higher in HRG | **** | 4-1BBL; complex TME role |
| CD70 | — | Not reported | Higher in HRG | *** | T-cell exhaustion; poor prognosis marker |
| PDCD1 | — | Not reported | Higher in HRG | * | PD-1; immune exhaustion |
| TNFSF15 | — | Not reported | Higher in LRG | **** | Anti-tumor immune activation |
| CD160 | — | Not reported | Higher in LRG | **** | NK/CD8+ T cell cytotoxicity |
| CD40LG | — | Not reported | Higher in LRG | *** | Anti-tumor immune activation |
| ADORA2A | — | Not reported | Higher in LRG | ** | Adenosine pathway; immunosuppression |
| IDO2 | — | Not reported | Higher in LRG | * | Metabolic immune modulator |

*Abbreviations: HRG, high-risk group; LRG, low-risk group; ICG, immune checkpoint gene; PIM, phosphoinositide metabolism; TME, tumor microenvironment.*
